# Early treatment and PD1 inhibition enhance HIV-specific functionality of follicular CD8^+^ T cells

**DOI:** 10.1172/jci.insight.180309

**Published:** 2025-04-08

**Authors:** Susanne Rueger, Eva Gruener, Danni Wang, Faiaz Shaik Abdool, Veronica Ober, Theresa Vallée, Renate Stirner, Raffaele Conca, Immanuel Andrä, Lisa Rogers, Robert Zahn, Elke Gersbacher, Joanna Eger, Ramona Pauli, Nils Postel, Christoph D. Spinner, Jörg J. Vehreschild, Melanie Stecher, Hans Nitschko, Josef Eberle, Johannes R. Bogner, Ulrich Seybold, Rika Draenert, Al Leslie, Henrik N. Kløverpris, Christof Geldmacher, Maximilian Muenchhoff, Kathrin Held, Julia Roider

**Affiliations:** 1 Department of Infectious Diseases, Department of Medicine IV, LMU University Hospital, LMU Munich, Munich, Germany.; 2 German Centre for Infection Research (DZIF), partner site Munich, Germany.; 3 Institute of Infectious Diseases and Tropical Medicine, LMU University Hospital, LMU Munich, Munich, Germany.; 4 Africa Health Research Institute (AHRI), and; 5 Department of Laboratory Medicine and Medical Science, Nelson R. Mandela School of Medicine, University of KwaZulu-Natal (UKZN), Durban, South Africa.; 6 Max von Pettenkofer Institute and Gene Center, Virology, National Reference Center for Retroviruses, and; 7 Department of Pediatrics, Dr. von Hauner Children’s Hospital, LMU University Hospital, LMU Munich, Munich, Germany.; 8 Institute for Medical Microbiology, Immunology and Hygiene, Technical University of Munich, Munich, Germany.; 9 Division of Transfusion Medicine, Cell Therapeutics and Haemostaseology, LMU University Hospital, LMU Munich, Munich, Germany.; 10 MUC Research, Clinical Research, Munich, Germany.; 11 Zentrum fuer Innere Medizin und Infektiologie, Munich, Germany.; 12 MVZ am Isartor, Munich, Germany.; 13 prinzmed, Practice for Infectiology, Munich, Germany.; 14 TUM School of Medicine and Health, Department of Clinical Medicine – Clinical Department for Internal Medicine II, University Medical Center, Technical University of Munich, Munich, Germany.; 15 Medical Department 2, Hematology/Oncology and Infectious Diseases, University Hospital of Frankfurt, Frankfurt, Germany.; 16 University of Cologne, Faculty of Medicine and University Hospital Cologne, Department I for Internal Medicine, Cologne, Germany.; 17 German Centre for Infection Research (DZIF), partner site Bonn-Cologne, Germany.; 18 Antibiotic Stewardship Team, LMU University Hospital, LMU Munich, Munich, Germany.; 19 Department of Infection and Immunity, University College London (UCL), London, United Kingdom.; 20 Department of Immunology and Microbiology, University of Copenhagen, Copenhagen, Denmark.; 21 Fraunhofer Institute for Translational Medicine and Pharmacology ITMP, Immunology, Infection and Pandemic Research, Munich, Germany.; 22 Unit Global Health, Helmholtz Zentrum München, German Research Centre for Environmental Health (HMGU), Neuherberg, Germany.

**Keywords:** AIDS/HIV, Immunology, Adaptive immunity, T cells

## Abstract

People living with HIV treated during acute infection are the group for whom achieving functional cure appears most viable. Follicular CD8^+^ T cells could contribute to HIV reservoir clearance by accessing B cell follicles through CXCR5 expression. This study examines peripheral follicular CD8^+^ T cells using flow cytometry, transcriptome analyses, and functional assays in people treated during acute (*n* = 37) and chronic (*n* = 18) infection, as well as in individuals naturally controlling HIV (*n* = 20) and living without HIV (*n* = 10). Our results reveal that early, as opposed to late, treatment initiation preserves antiviral effector functions of follicular CD8^+^ T cells, which are further enhanced by PD1 inhibition. We also identify a correlation between follicular CD8^+^ T cells and intact proviral HIV DNA levels in acute, but not chronic, infection. Longitudinal transcriptomic analysis of peripheral effector cells after 48 weeks of suppressive therapy indicated traits of recent antigen exposure, suggesting potential recirculation into lymphoid tissue. These findings underscore the pivotal role of follicular CD8^+^ T cells in anti-HIV responses and support investigating targeted cure strategies, such as anti-PD1 therapy, especially in individuals initiating treatment during acute infection.

## Abstract 2

<span>Circulating follicular CD8 T cells from individuals treated in early HIV infection show antiviral effector functions and PD1 blockade enhances their vigorous HIV-specific functionality.</span>

## Introduction

Lymphoid tissues are an important site for residual active and latent HIV/SIV infection persisting despite antiretroviral therapy (ART) (1–4) and natural immunity (5–8). This viral reservoir poses a substantial challenge to HIV cure, as it fuels rapid rebound of plasma viremia during ART interruption (9, 10).

Evidence highlights the central role of CD8^+^ T cells in controlling HIV/SIV infection (11–20), including in individuals showing spontaneous viral control (8, 11, 21, 22). To control viral replication, HIV-specific CD8^+^ T cells must be able to traffic to and function at the anatomical sites where HIV resides, such as germinal centers (GCs) within lymphoid B cell follicles (BCFs). HIV/SIV-infected CD4^+^ T cells persist in GCs even during ART or in elite controllers (5–8, 23–27). Conventional CD8^+^ T cells are largely excluded from BCFs (28–30), where noncytolytic CD8^+^ T cells predominate (28, 29, 31), which may represent a substantial obstacle to CD8^+^ T cell–mediated clearance of the HIV-infected follicular helper T cell reservoir (5, 32).

Follicular CD8^+^ T cells express the follicle-homing receptor CXCR5 for BCF trafficking, where they could eliminate HIV-infected cells within these reservoir sites (1, 33–35). Indeed, viral control has been associated with a higher proportion and functionality of CXCR5^+^CD8^+^ T cells in circulation and in lymphoid tissue (8, 36, 37). Yet, the transcriptional network driving CXCR5 expression on CD8^+^ T cells, and therefore entry into BCFs, reduces cytotoxic function and induces a memory-like phenotype, resulting in compromised immunosurveillance (34, 38). Additionally, CXCR5^+^CD8^+^ T cells express elevated levels of programmed cell death protein 1 (PD1), associated with immune exhaustion in chronic viral infections including HIV (39–41).

These observations underscore the need to develop strategies that promote not only follicular entry of CD8^+^ T cells but also enhance cytotoxic effector functions of HIV-specific CXCR5^+^CD8^+^ T cells. Cancer immunotherapy principles (42) could be applied to HIV, such as PD1/PD-L1 blockade, which improves HIV-specific immunity in vitro (41) and in vivo (43, 44). Moreover, a small animal study of chronic viral infection demonstrated that the proliferative burst after PD1 blockade originated from virus-specific CXCR5^+^CD8^+^ T cells (45), with potential implications for HIV infection.

ART initiation during primary HIV infection is thought to reduce HIV reservoir size (46–49). While attempts to completely prevent the establishment of latency through the use of early ART in nonhuman primates (50, 51) or in humans (9, 52) have been unsuccessful, rare cases of individuals controlling HIV after cessation of ART (“posttreatment controllers”) (53, 54) indicate that early ART may facilitate long-term HIV control and functional cure (53, 55).

Here, we longitudinally study how ART initiation during acute infection (Fiebig I–IV) shapes follicular phenotype and HIV-specific effector functions of circulating CXCR5^+^CD8^+^ T cells as well as the HIV reservoir, using individuals treated during chronic infection and those who naturally control HIV as controls. Using flow cytometry, transcriptome analyses of these rare CXCR5^+^ HIV-specific cells, and functional assays, we show that initiating ART early, as opposed to late, effectively preserves antiviral effector functions in CXCR5^+^CD8^+^ T cells and that their HIV-specific cytotoxicity is boosted by PD1 blockade in vitro. These findings carry important implications for future clinical trials exploring functional cure of HIV, especially in individuals treated early during infection.

## Results

### Circulating CXCR5^+^CD8^+^ T cells coexpress PD1 and CD127.

**Figure 1:**
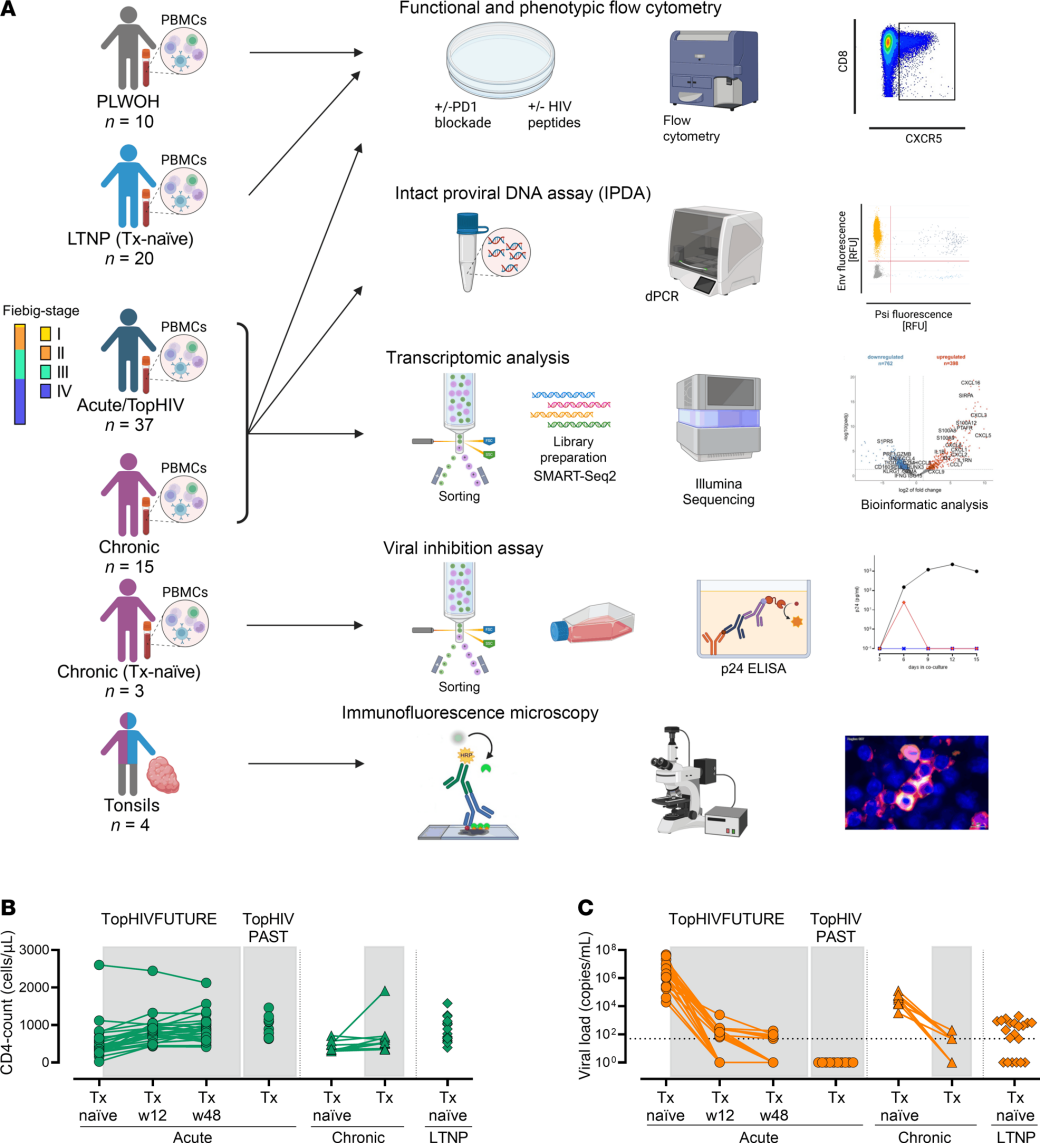
Study design and progress-describing parameters. (**A**) Overview of the study design. PLWOH, people living without HIV; LTNP, individuals naturally controlling HIV; Acute, individuals treated during acute HIV infection; Chronic, individuals treated during chronic HIV infection; Tonsils, individuals living with or without HIV and donating tonsils; Tx, treatment. Created in BioRender; https://BioRender.com/i33u513 (**B** and **C**) Longitudinal and individual CD4^+^ counts (**B**) and plasma viral load (**C**) in peripheral blood of participants analyzed by flow cytometry. The dotted line indicates the detection limit (<50 copies/mL). Time points on ART are shaded in gray.

**Figure 2:**
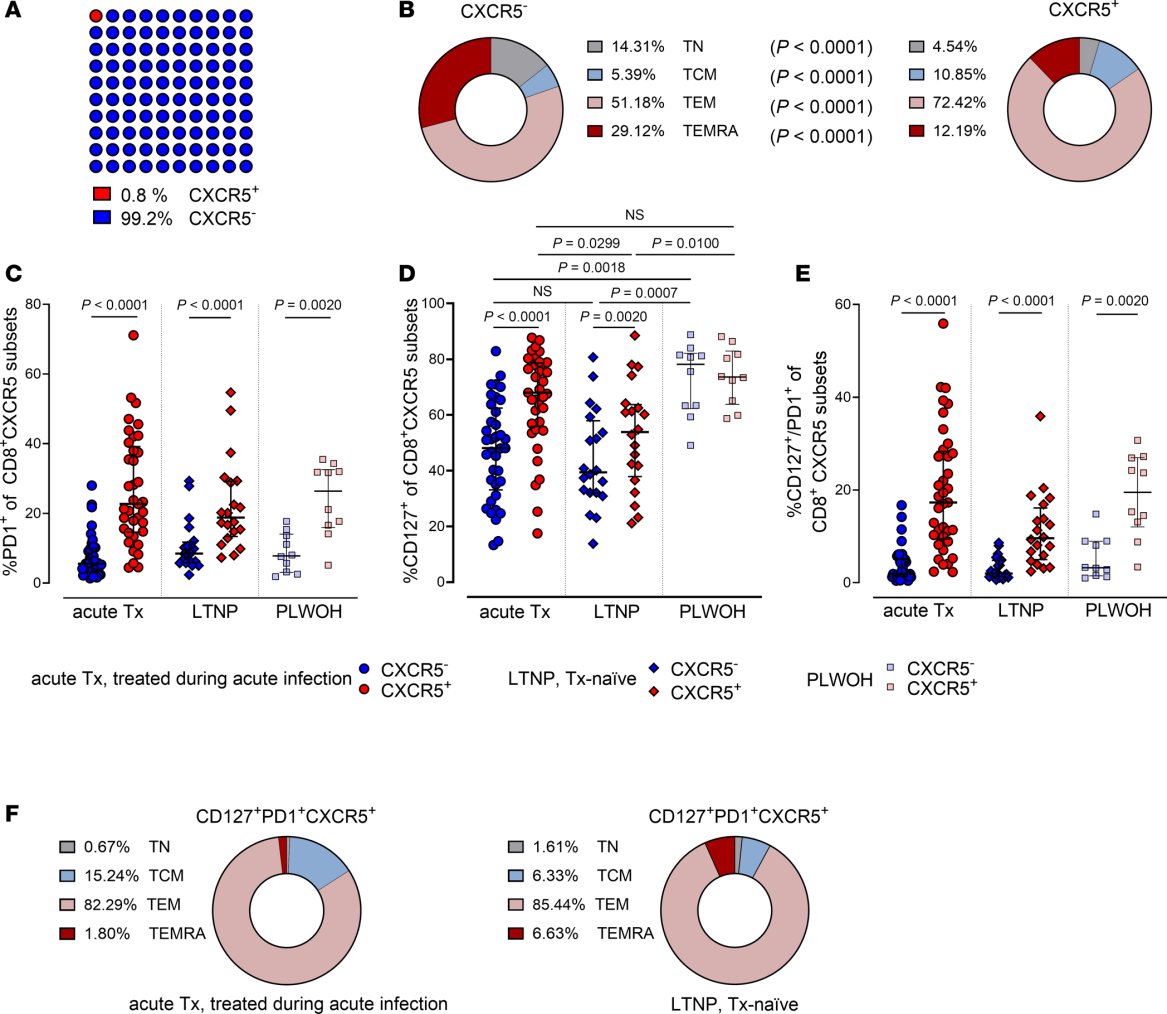
Circulating CXCR5^+^CD8^+^ T cells coexpress PD1 and CD127. (**A**) Frequency of CXCR5^+^CD8^+^ T cells in peripheral blood of individuals treated during acute infection (*n* = 37; median 52 weeks on ART). Red, follicular (CXCR5^+^); blue, nonfollicular (CXCR5^–^). (**B**) Memory subset distribution stratified by CD45RA and CCR7 expression in CXCR5^+^CD8^+^ versus CXCR5^–^CD8^+^ T cells in the same group as **A**. Median values were adjusted to the total, defined as 100%. *P* values by Wilcoxon’s matched-pairs signed-rank test. (**C**–**E**) Frequencies of CXCR5^–^ (blue) and CXCR5^+^ (red) CD8^+^ T cells expressing PD1 (**C**), CD127 (**D**), and coexpressing PD1/CD127 (**E**) in individuals treated during acute infection (*n* = 37; circles; median 52 weeks on ART), in individuals naturally controlling HIV (LTNPs; *n* = 20; diamonds), and in people LWOH (PLWOH; *n* = 10; squares). *P* values by Wilcoxon’s matched-pairs signed-rank test (within 1 group) and Kruskal-Wallis test with Dunn’s correction for multiple comparisons (between groups). Medians and IQRs are indicated. (**F**) Memory subset distribution stratified by CD45RA/CCR7 expression in CD127^+^PD1^+^CXCR5^+^CD8^+^ T cells of individuals treated during acute infection (*n* = 37; median 52 weeks on ART) and individuals naturally controlling HIV (LTNPs; *n* = 20). Median values were adjusted to the total, defined as 100%. TN, naive T cell; TCM, central memory T cell; TEM, effector memory T cell; TEMRA, effector memory T cells re-expressing CD45RA.

CXCR5^+^CD8^+^ T cells have been described as stem-like cells (45, 56) with increased T helper (57–59) and reduced cytotoxic functions (8, 28, 35) compared with CXCR5^–^CD8^+^ T cells. We aimed to characterize these cells in people living with HIV (LWH), focusing on 2 cohorts relevant for HIV cure: (a) individuals treated during acute infection (Fiebig I–IV) and (b) individuals naturally controlling HIV (“LTNPs”), with people treated during chronic infection and living without HIV (LWOH) serving as controls (Table 1, Supplemental Table 1, Figure 1, and Supplemental Figure 1; supplemental material available online with this article; https://doi.org/10.1172/jci.insight.180309DS1). Confirming previous results (60), circulating CXCR5^+^CD8^+^ T cell frequency was small (0.8% of all CD8^+^ cells; Figure 2A), predominantly differentiated in an effector memory (CD45RA^–^CCR7^–^) phenotype (72.4% on CXCR5^+^ vs. 51.2% on CXCR5^–^, *P* < 0.0001; Figure 2B and Supplemental Figure 2A for gating strategy). CXCR5^+^CD8^+^ T cells, regardless of HIV status, expressed significantly more PD1 than CXCR5^–^CD8^+^ T cells (Figure 2C and Supplemental Figure 2B), as described previously (1, 37, 45, 61). The well-described HIV-associated downregulation of the IL-7 receptor CD127 on CD8^+^ T cells (62–64) was not observed on CXCR5^+^CD8^+^ T cells of individuals treated during acute infection and less prominent on CXCR5^+^CD8^+^ T cells across all groups LWH (Figure 2D and Supplemental Figure 2C). Furthermore, approximately 20% of CXCR5^+^CD8^+^ T cells coexpressed CD127 and PD1, independently of HIV infection. This phenotype was absent in their CXCR5^–^ counterparts (Figure 2E and Supplemental Figure 2D). PD1^+^CD127^+^CXCR5^+^CD8^+^ T cells were mainly differentiated into an effector memory phenotype (Figure 2F).

### CXCR5^+^CD8^+^ T cells are located inside and outside of tonsil GCs and show a distinct transcriptomic profile in circulation compared with CXCR5^–^CD8^+^ T cells.

**Figure 3:**
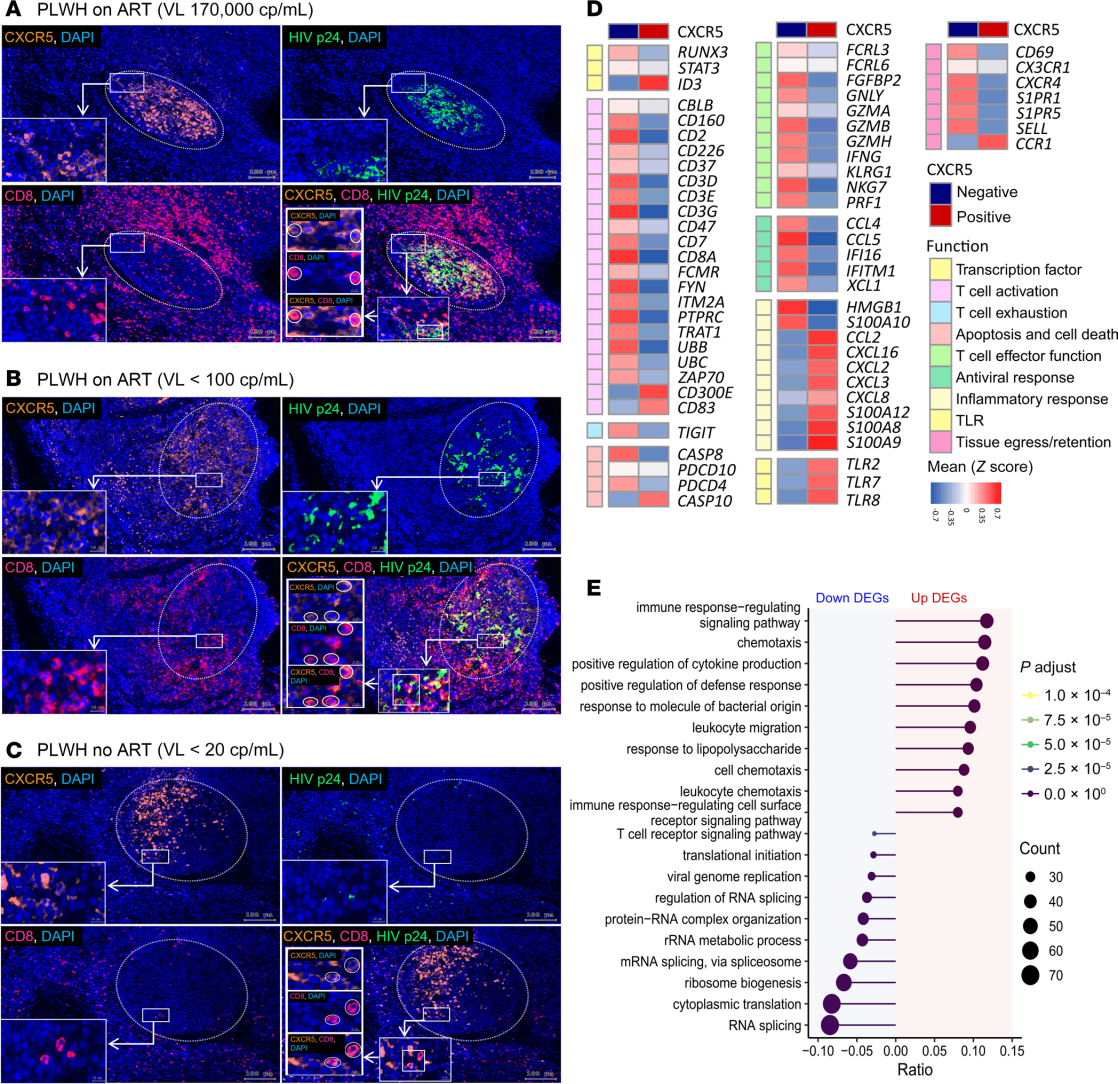
CXCR5^+^CD8^+^ T cell localization in tonsils and their distinct transcriptomic profile in circulation compared with CXCR5^–^CD8^+^ T cells. (**A**–**C**) Immunofluorescence microscopy of tonsil tissue from participants LWH: viremic (**A**) and aviremic participant on ART (**B**) and an individual naturally controlling HIV (**C**). VL, viral load; cp, copies. Whole tonsil tissue sections were stained, and GCs identified (white dotted circles). Inserts represent zoomed into areas of GCs (white rectangles). Orange, CXCR5; pink, CD8; green, HIV p24; blue, DAPI/nuclear counterstain. Original magnification, ×40. Scale bars: 100 μm, 10 μm (insets), and 5 μm (zoomed-in insets). See Supplemental Figure 3C for the isotype control staining. (**D**) Heatmap of selected differentially expressed genes (DEGs) in CXCR5^+^CD8^+^ versus CXCR5^–^CD8^+^ T cells from people LWH (*n* = 13; max 4 weeks on ART) categorized in functional gene groups. Relative changes in gene expression between CXCR5^+^CD8^+^ and CXCR5^–^CD8^+^ T cells are depicted. Normalized read counts, calculated with DESeq2, were converted into *z* scores and mean *z* scores of all analyzed individuals per gene per CXCR5^+^ and CXCR5^–^ cell types are plotted. Plotted DEGs were selected based on their function. Red indicates higher and blue lower expression. (**E**) Enriched GO terms (biological process) from significantly DEGs that are downregulated (left, blue background) and upregulated (right, red background) in CXCR5^+^CD8^+^ T cells compared with their CXCR5^–^ counterparts (BH-adjusted *P* values < 1 × 10^–4^). The line length represents the ratio of the number of genes that are part of the pathway differentially expressed between cell types to the total number of genes differentially expressed between cell types. Dot size represents the absolute observed number of DEGs in CXCR5^+^CD8^+^ versus CXCR5^–^CD8^+^ T cells per pathway. Line and dot color code is according to the adjusted *P* value.

As demonstrated previously (38, 60), CXCR5^+^CD8^+^ T cells are found both inside and outside of GCs of BCFs (Figure 3, A–C), as well as in circulation in people LWH (Figure 2A) (6, 34, 38, 65). Quantification revealed increased tonsil GC CD8^+^ T cell frequencies in viremic infection (*P* = 0.0021, viremic vs. without HIV; Supplemental Figure 3, A–C) (1). In line with this, our transcriptome analyses revealed a downregulation of tissue-resident (*CD69*) and lymph node–homing markers (*S1PR1/5*, *SELL*) (66) (Figure 3D) in circulating CXCR5^+^CD8^+^ versus CXCR5^–^CD8^+^ T cells. Supporting our flow cytometry findings of a dominant effector memory phenotype in CXCR5^+^CD8^+^ T cells (Figure 2B), we observed clear differential regulation of the transcription factors *ID3* (upregulated) and *RUNX3* (downregulated) in CXCR5^+^ cells, corroborating the follicular phenotype (67, 68). Furthermore, CXCR5^+^CD8^+^ T cells, compared with their CXCR5^–^ counterparts, exhibit a marked downregulation of genes associated with T cell activation (e.g., costimulatory molecule *CD2*, activating receptor *CD226*, molecules involved in T cell receptor [TCR] signaling like *CD3* components and *ZAP70*) and T cell exhaustion (the inhibitory checkpoint receptor *TIGIT*), confirming previous results (68, 69). Aptly, gene set enrichment analysis (GSEA) confirmed that an exhaustion gene set (70) was enriched among CXCR5^–^CD8^+^ but not CXCR5^+^CD8^+^ T cells (Supplemental Figure 3D). Apoptotic genes (e.g., *CASP8*, *PDCD4*) were downregulated, suggesting a better survival potential of CXCR5^+^CD8^+^ T cells, as reported in SIV-specific CXCR5^+^ T cells in infected macaques (71), in line with our flow cytometry data (Figure 2D). Unlike CXCR5^–^CD8^+^, CXCR5^+^CD8^+^ T cells transcribed less of the classical T cell effector genes, with downregulation of transcripts encoding (cytolytic) effector molecules (e.g., *GZMA*, *GZMB*, *GNLY*, *IFNG*, *PRF1*). Interestingly, innate IFN-stimulated genes (e.g., *IFI16*, *IFITM*) were downregulated but expression of several Toll-like receptors (TLRs; *TLR2*, *TLR7*, *TLR8*) and inflammatory cytokines and chemokines (e.g., *S100A8/9/10/12*, *CCL2*, *CXCL2/3/8/16*) were upregulated in CXCR5^+^CD8^+^ T cells, an observation that we confirmed by flow cytometry for some of the identified genes (*TLR2*, *TLR8*; Supplemental Figure 3E). Gene Ontology (GO) enrichment analysis revealed that upregulated differentially expressed genes (DEGs) in CXCR5^+^CD8^+^ T cells are linked to immune response, cytokine production, defense response, and cell chemotaxis, while downregulated DEGs are linked to TCR signalling pathways (Figure 3E). Overall, CXCR5^+^CD8^+^ T cells showed a downregulation of genes associated with cytotoxic T cell effector functions, T cell activation, and exhaustion when compared with CXCR5^–^CD8^+^ T cells, while simultaneously upregulating cytokine expression as well as genes associated with innate inflammatory and TLR responses (Figure 3D and Supplemental Figure 3F).

### Circulating HIV-specific CXCR5^+^CD8^+^ T cells show a classical follicular, non–tissue-resident-like transcriptomic profile.

**Figure 4:**
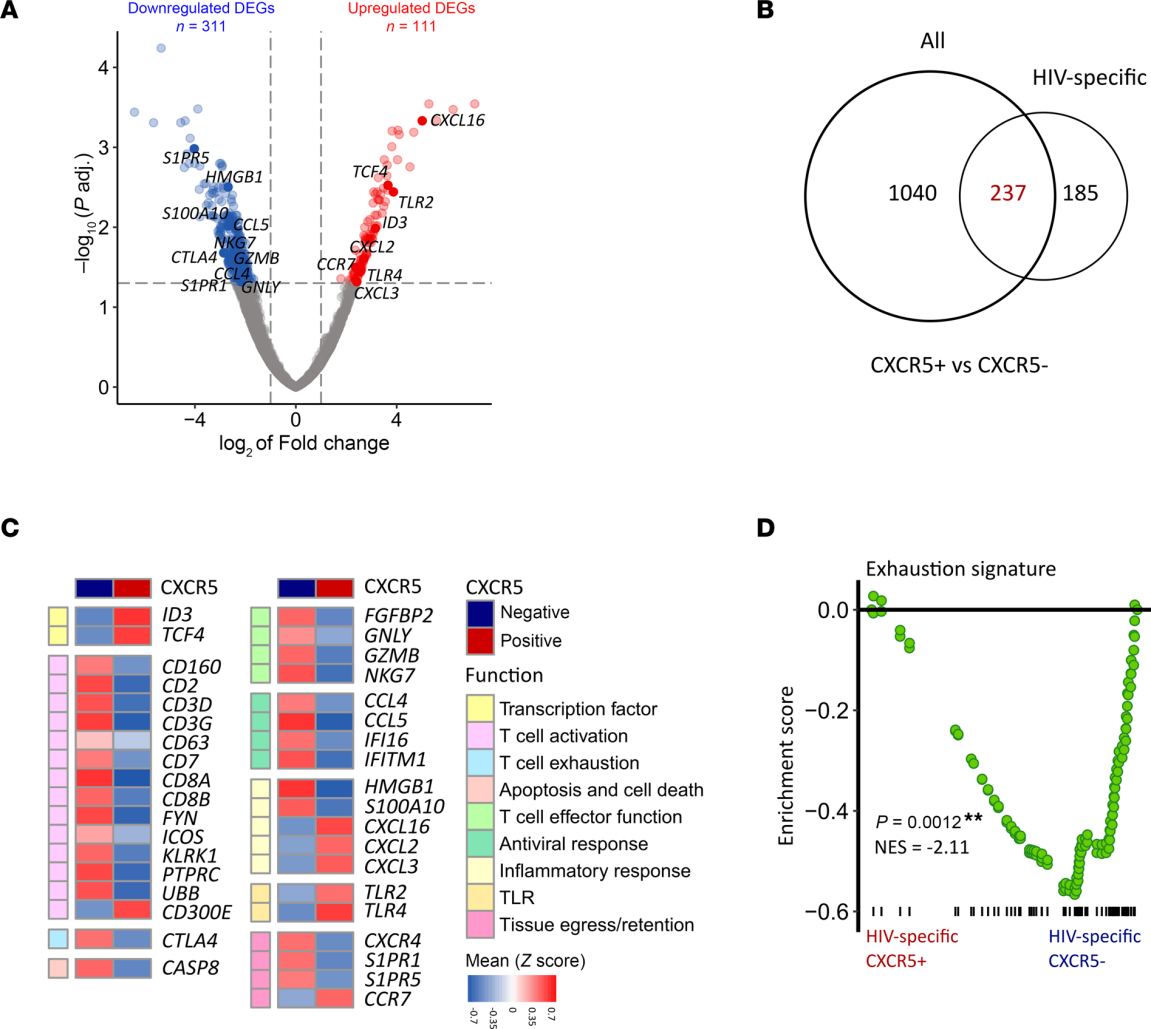
Circulating HIV-specific CXCR5^+^CD8^+^ T cells show a classical follicular, non–tissue-resident-like transcriptomic profile. (**A**) Volcano plot depicting upregulated (red) and downregulated (blue) DEGs in HIV-specific CXCR5^+^CD8^+^ versus CXCR5^–^CD8^+^ T cells. Cutoffs for differential expression: adjusted *P* value < 0.05 and absolute log_2_(fold-change) > 1. Selected immune-related genes are highlighted. (**B**) Number of genes significantly differentially regulated in bulk and HIV-specific CXCR5^+^CD8^+^ versus CXCR5^–^CD8^+^ T cells, with overlapping DEGs in red. (**C**) Heatmap of selected DEGs in HIV-specific CXCR5^+^CD8^+^ versus CXCR5^–^CD8^+^ T cells categorized in functional groups. Relative changes in gene expression between tetramer-binding CXCR5^+^CD8^+^ and CXCR5^–^CD8^+^ T cells are depicted. Normalized read counts, calculated with DESeq2, were converted into *z* scores and mean *z* scores per gene per HIV-specific CXCR5^+^ and CXCR5^–^ cell types are plotted. Plotted DEGs were selected based on their function. Red represents higher and blue lower gene expression. (**D**) GSEA shows exhaustion signature (70) enrichment in HIV-specific CXCR5^–^CD8^+^ but not CXCR5^+^CD8^+^ T cells. (**A**–**D**) CXCR5^+^CD8^+^ T cells were sorted from people LWH (*n* = 13; max 4 weeks on ART). NES, normalized enrichment score.

To investigate whether HIV specificity, defined by tetramer positivity (A*02:01-restricted HIV gag SL9, ref. 72; B*40:02-restricted HIV nef KL9, refs. 73, 74; or B*07:02-restricted HIV nef RM9, ref. 75; Supplemental Table 1), changes the follicular phenotype, we analyzed differential gene expression in HIV-specific tetramer^+^ (Tet^+^) versus unselected Tet^–^ CD8^+^ T cells (Figure 4A). Among 422 DEGs identified, more than 50% (*n* = 237) overlapped with DEGs found in CXCR5^+^CD8^+^ versus CXCR5^–^CD8^+^ T cells (Figure 4B). The remaining DEGs (*n* = 185) are related to cell skeleton remodelling and basic cellular functions. In detail, like bulk Tet^–^CXCR5^+^CD8^+^ T cells, HIV-specific Tet^+^CXCR5^+^CD8^+^ T cells showed lower expression of genes for T cell activation (e.g., *CD2*, *CD3D/G*, *ICOS*, *KLRK1* [*NKD2D*]) and exhaustion (*CTLA4*) compared with their CXCR5^–^ counterparts (Figure 4C). Like bulk CXCR5^+^CD8^+^ T cells, HIV-specific Tet^+^CXCR5^+^CD8^+^ T cells downregulated *GZMB* and *GNLY*, genes encoding cytolytic effector molecules, and several innate antiviral genes (e.g., *CCL4/5*, *IFI16*), while upregulating genes associated with inflammatory response (e.g., *CXCL2/3/16*, *TLR2/4*). One proapoptotic gene (*CASP8*) was also downregulated in HIV-specific Tet^+^CXCR5^+^CD8^+^ T cells. Moreover, again like bulk Tet^–^CXCR5^+^CD8^+^ T cells (Figure 3D), tissue egress and lymph node–homing markers (*S1PR1/5*) were downregulated in HIV-specific Tet^+^CXCR5^+^CD8^+^ T cells (Figure 4C). Interestingly, the lymph node–homing marker *CCR7* was upregulated in these cells (Figure 4C).

To ascribe functional states to circulating HIV-specific Tet^+^CXCR5^+^CD8^+^ T cells, we performed GSEA using published gene sets derived from (a) HIV-specific tissue-resident memory CD8^+^ T cells from human lymph nodes (30), (b) exhausted T cells (70), (c) T cell proliferation, and (d) T cell activation (76, 77) in our cellular gene expression data. In line with findings in bulk CXCR5^+^CD8^+^ T cells (Supplemental Figure 3D), GSEA confirmed an exhaustion phenotype in HIV-specific CXCR5^–^CD8^+^ but not CXCR5^+^CD8^+^ T cells (Figure 4D).

Taken together, these results show that circulating HIV-specific Tet^+^CXCR5^+^CD8^+^ T cells mirror the low activation and exhaustion pattern of bulk CXCR5^+^CD8^+^ T cells with downregulation of genes associated with cytotoxic effector functions, while simultaneously upregulating genes associated with inflammatory and TLR responses. It needs to be stated, however, that the used HIV tetramers only characterize a small subset of antiviral T cells, and the group of bulk CXCR5^+^CD8^+^ T cells presumably contains many antiviral cells with heterogeneous antigen specificities.

### Circulating CXCR5^+^CD8^+^ T cells exhibit HIV-specific effector functions upon antigen stimulation.

**Figure 5:**
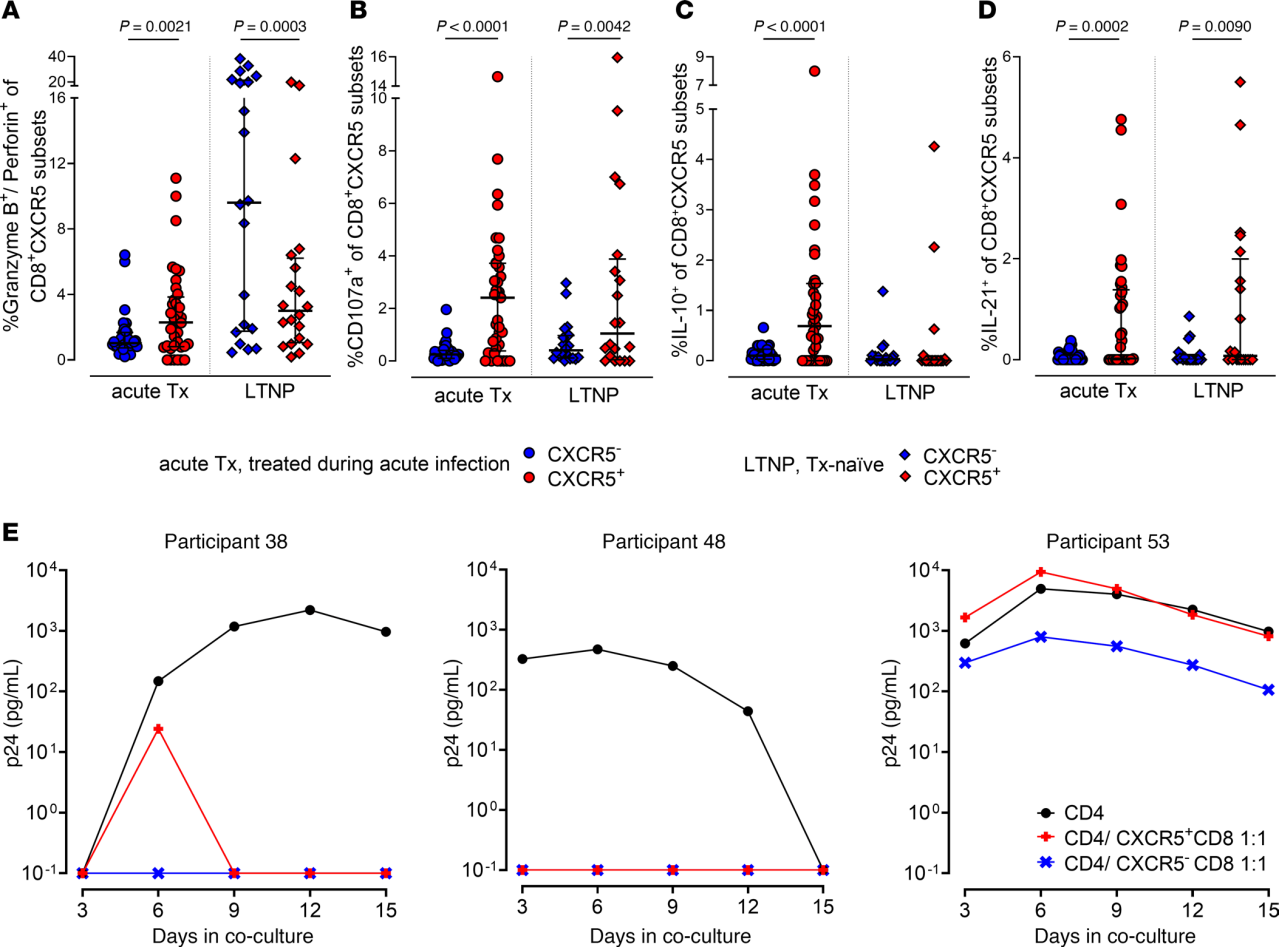
Circulating CXCR5^+^CD8^+^ T cells exhibit HIV-specific effector functions upon antigen stimulation. (**A**–**D**) Frequencies of CXCR5^–^CD8^+^ (blue) and CXCR5^+^CD8^+^ (red) T cells coexpressing granzyme B and perforin ex vivo (**A**), and CD107a (**B**), IL-10 (**C**), or IL-21 (**D**) after HIV gag peptide stimulation in individuals treated during acute infection (*n* = 37; circles; median 52 weeks on ART) and individuals naturally controlling HIV (LTNPs; *n* = 20; diamonds). *P* values by Wilcoxon’s matched-pairs signed-rank test. Medians and IQRs are indicated. (**E**) HIV p24 concentration in culture supernatants of PHA-activated CD4^+^ T cells from 3 viremic individuals without (black) or with autologous, unstimulated CXCR5^–^CD8^+^ (blue) or CXCR5^+^CD8^+^ (red) T cells (effector/target ratio 1:1) (Supplemental Table 2).

To assess HIV-specific effector functions of CXCR5^+^CD8^+^ versus CXCR5^–^CD8^+^ T cells, PBMCs from 3 clinical groups of people LWH were stimulated in vitro with an HIV gag peptide pool and intracellularly stained for effector molecules. Surprisingly, CXCR5^+^CD8^+^ T cells displayed a strong cytotoxic phenotype in individuals treated during acute infection or naturally controlling HIV (Figure 5A), but not in people treated during chronic infection (Supplemental Figure 4A). These cells exhibited higher degranulation in response to HIV gag stimulation when compared with their CXCR5^–^ counterparts (*P* < 0.0001; Figure 5B and Supplemental Figure 4A). Consistent with findings of others (78, 79), the enrichment of HIV-specific cytokine secretion by CXCR5^+^CD8^+^ T cells was not limited to cytotoxicity but also observed for IL-10 (*P* < 0.0001; Figure 5C) and IL-21 (*P* = 0.0002; Figure 5D). Subgroup analyses revealed that PD1^+^CD8^+^ T cells, regardless of CXCR5 (Supplemental Figure 4B) or CD127 (Supplemental Figure 4C) (co)expression, secreted significantly less cytotoxic cytokines compared with their PD1^–^ counterparts.

To evaluate the functional relevance of these findings, we assessed the ability of CXCR5^+^CD8^+^ T cells to control viral replication in vitro. We observed a comparable inhibition of autologous virus replication between CXCR5^+^CD8^+^ and CXCR5^–^CD8^+^ T cells in 2 out of 3 treatment-naive individuals LWH (Figure 5E and Supplemental Table 2). In the third individual, neither subset controlled viral growth.

Overall, stimulation of CD8^+^ T cells with HIV antigen — either by gag peptides or virus — revealed HIV-specific functionality of CXCR5^+^CD8^+^ T cells characterized by upregulation of cytotoxic effector molecules, degranulation markers, cytokine secretion, and viral control.

### Circulating CXCR5^+^CD8^+^ T cells inversely correlate with intact proviral HIV DNA after 48 weeks of ART in individuals treated during acute infection.

**Figure 6:**
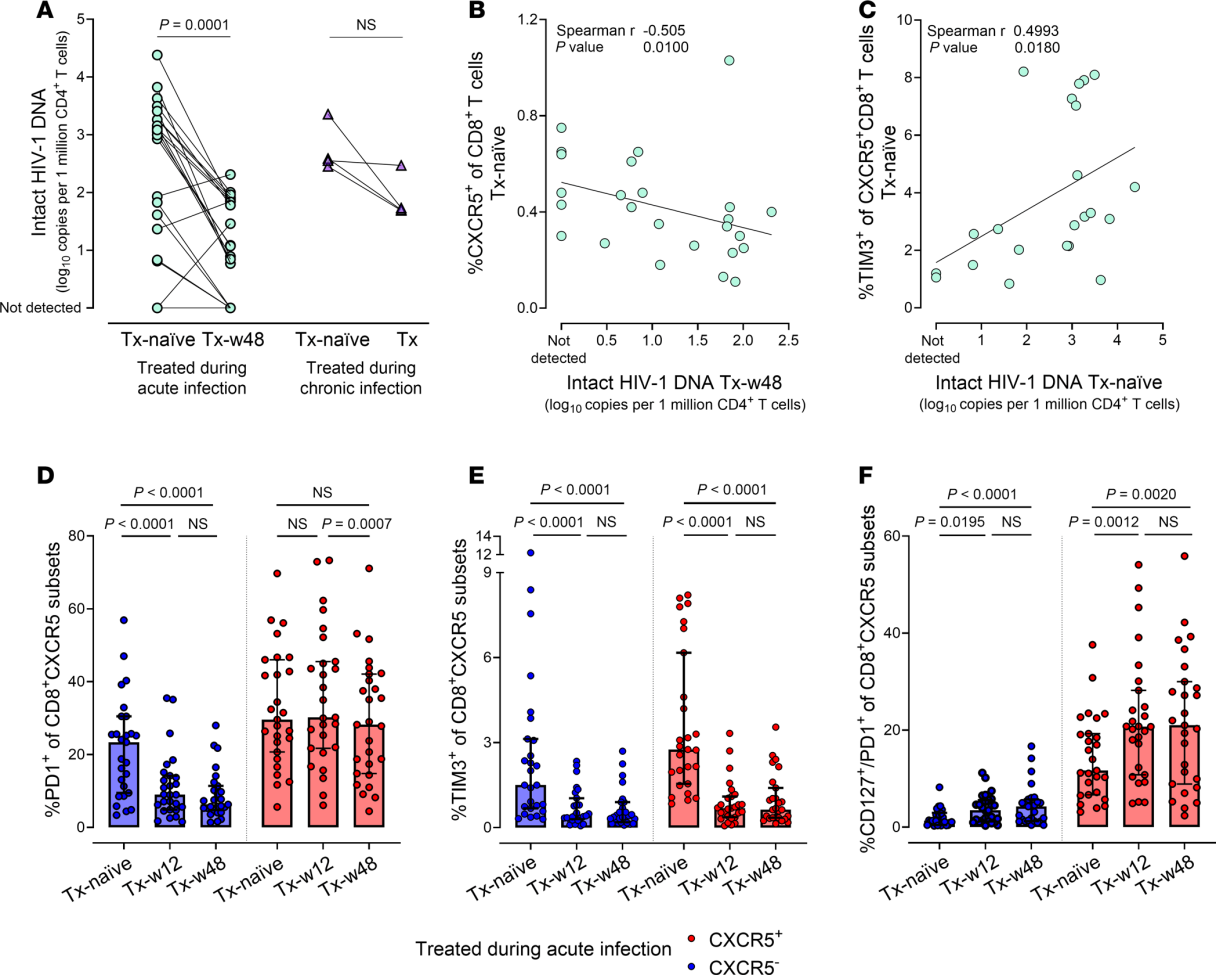
Circulating CXCR5^+^CD8^+^ T cells inversely correlate with intact proviral HIV DNA after 48 weeks of ART in individuals treated during acute infection. (**A**) Intact proviral HIV DNA levels in CD4^+^ T cells from individuals with acute (TopHIVFUTURE; *n* = 21, green circles) and chronic HIV infection (*n* = 4, purple triangles) before and during ART. *P* values by Wilcoxon’s matched-pairs signed-rank test. (**B**) Negative correlation between CXCR5^+^CD8^+^ T cell frequency prior to ART and intact proviral HIV DNA after 48 weeks of ART in individuals treated during acute infection (TopHIVFUTURE; *n* = 25). Spearman’s rank correlation was performed. (**C**) Spearman’s rank correlation between TIM3^+^CXCR5^+^CD8^+^ T cell frequency and intact proviral HIV DNA in individuals treated during acute infection (TopHIVFUTURE; *n* = 22) prior to ART. (**D**–**F**) Longitudinal analysis of PD1 (**D**), TIM3 (**E**), and CD127/PD1 (**F**) (co)expression on CXCR5^–^CD8^+^ (blue) and CXCR5^+^CD8^+^ (red) T cells in individuals treated during acute infection (TopHIVFUTURE; *n* = 27). *P* values by Friedman’s test with Dunn’s correction for multiple comparisons.

Most HIV proviruses persisting under ART are defective (80–82). Therefore, the intact proviral DNA fraction represents the more accurate correlate of the viral reservoir (80, 83). This observation holds true in our cohort, with defective proviruses predominating under ART (Supplemental Figure 5A). We further observed a significant decline of intact proviral HIV DNA after 48 weeks of early ART (Figure 6A and Supplemental Figure 5A). Importantly, baseline CXCR5^+^CD8^+^ T cell frequency correlated negatively with intact proviral HIV DNA levels under ART in individuals treated during acute (*r*_S_ *=* –0.51, *P* = 0.01; Figure 6B), but not chronic (Supplemental Figure 5, B and C) infection. Furthermore, intact proviral HIV DNA loads correlated positively with TIM3^+^CXCR5^+^CD8^+^ T cell frequencies prior to ART (Figure 6C).

PD1^+^CXCR5^+^CD8^+^ T cell frequencies remained unchanged after early ART (Figure 6D), in contrast with the significant changes observed after late ART, or in CXCR5^–^CD8^+^ T cells, or regarding TIM3 expression after early ART (Figure 6, D and E, and Supplemental Figure 5, D and E). Early and late ART initiation led to a significant increase in both CD127^+^CXCR5^+^CD8^+^ and PD1^+^CD127^+^CXCR5^+^CD8^+^ T cells (Figure 6F and Supplemental Figure 5, F–H).

In conclusion, our findings suggest that CXCR5^+^CD8^+^ T cells contribute to viral control in individuals treated during acute, but not chronic infection, as shown by correlations between CXCR5^+^CD8^+^ T cells and intact proviral HIV DNA loads, an estimator for the viral reservoir. Crucially, early treatment not only reverses the exhausted phenotype of CD8^+^ T cells but also augments the percentage of CXCR5^+^CD8^+^ T cells with proliferative potential.

### ART initiation during acute HIV infection preserves HIV-specific effector functionality in CXCR5^+^CD8^+^ T cells.

**Figure 7:**
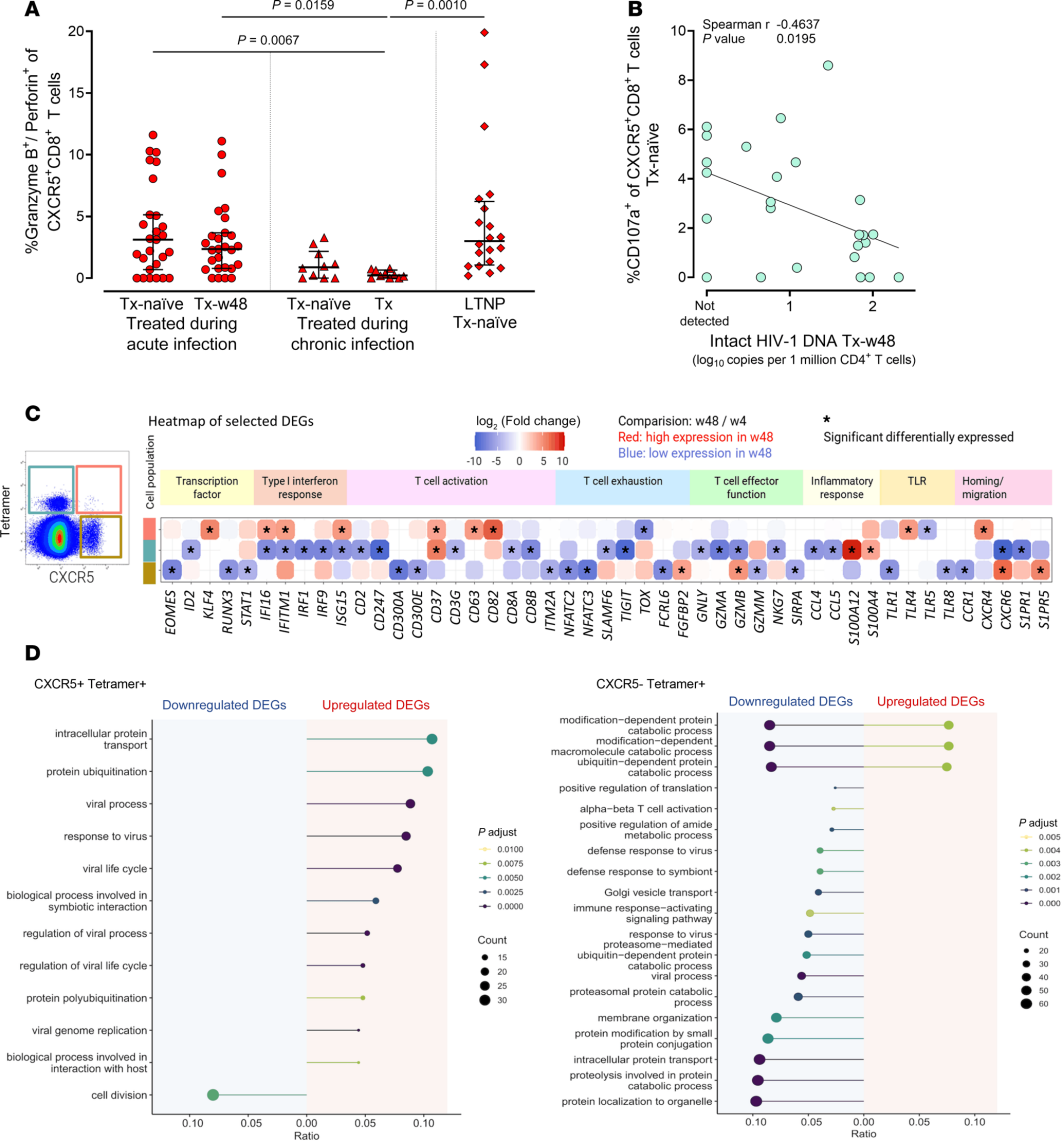
ART initiation during acute HIV infection preserves HIV-specific effector functionality in CXCR5^+^CD8^+^ T cells. (**A**) Frequencies of cytotoxic (granzyme B^+^perforin^+^) CXCR5^+^CD8^+^ T cells in individuals with acute (TopHIVFUTURE: *n* = 27; circles) and chronic (*n* = 10; triangles) HIV infection prior and during ART as well as in individuals naturally controlling HIV (LTNPs; *n* = 20; diamonds) ex vivo. *P* values by Kruskal-Wallis test with Dunn’s correction for multiple comparisons. (**B**) Correlation between HIV-specific CD107a expression on CXCR5^+^CD8^+^ T cells responding to overnight stimulation with HIV gag peptide pool at baseline and intact proviral HIV DNA after 48 weeks of ART in individuals treated during acute infection (TopHIVFUTURE; *n* = 25). Spearman’s rank correlation was performed. (**C**) Heatmap of genes significantly differentially regulated in individuals treated during acute HIV infection (*n* = 4; week 4 [w4] versus week 48 [w48] after ART initiation) in HIV-specific CXCR5^+^ and CXCR5^–^ cells as well as tetramer-negative CXCR5^+^CD8^+^ T cells categorized in functional groups. Relative changes in gene expression between w4 and w48 on ART depicted for each cell type. Normalized read counts, calculated with DESeq2, were converted into *z* scores and mean *z* scores per gene significantly differentially expressed across the time points are plotted. Plotted DEGs were selected based on their function. Red indicates higher and blue lower expression. (**D**) Enriched GO terms (biological process) for significantly downregulated (left) and upregulated (right) genes in HIV-specific CXCR5^+^CD8^+^ and CXCR5^–^CD8^+^ T cells w4 versus w48 after ART initiation (BH-adjusted *P* values < 0.01). The line length represents the ratio of the number of observed DEGs per pathway to the total number of DEGs, and dot size represents the absolute observed number of DEGs in CXCR5^+^CD8^+^ versus CXCR5^–^CD8^+^ T cells per pathway. Line and dot color code is according to the adjusted *P* value.

Having observed HIV-specific effector functions in circulating CXCR5^+^CD8^+^ T cells upon in vitro stimulation, we examined whether and how early ART affects this virus-specific functionality. Early treatment led to the preservation of granzyme B and perforin responses of CXCR5^+^CD8^+^ T cells over 48 weeks of ART, an effect absent in individuals treated during chronic infection (Figure 7A). Importantly, cytotoxic CXCR5^+^CD8^+^ T cell frequencies were comparable between individuals treated during acute infection and those naturally controlling HIV (“LTNP,” median 2.36% and 3.01%), and significantly higher compared with individuals treated during chronic infection (Figure 7A). In contrast, the strong gag-specific degranulation (measured by CD107a; Figure 5B) was unaffected by the timing of treatment initiation (Supplemental Figure 6A). Importantly, the frequency of HIV-specific CD107a-expressing CXCR5^+^CD8^+^ T cells at baseline correlated negatively with intact proviral HIV DNA levels after ART (Figure 7B and Supplemental Figure 6B).

To further understand the preservation of effector functions of HIV-specific CXCR5^+^CD8^+^ T cells by early ART, we studied longitudinal transcriptomic changes in HIV-specific Tet^+^ cells versus their CXCR5^–^ and Tet^–^ (mostly non–HIV-specific) counterparts. Longitudinal analyses of sorted memory CD8^+^ T cell populations (CXCR5^+^Tet^+^, CXCR5^+^Tet^–^, and CXCR5^–^Tet^+^) in individuals treated during acute infection (*n* = 4, Tx-w4 vs. Tx-w48) revealed unique transcriptomic changes after 48 weeks of ART for each cell population (Figure 7C). We observed that, in contrast with the changes in CXCR5^–^, CXCR5^+^ HIV-specific Tet^+^ CD8^+^ T cells increased their IFN responsiveness under prolonged suppressive ART, shown by the upregulation of numerous IFN-induced genes at the late time point. This upregulation of IFN-regulated genes was not observed in the bulk (Tet^–^) CXCR5^+^CD8^+^ T cell population. Focusing on genes involved in T cell activation, we observed an upregulation of the costimulatory molecule *CD82* in HIV-specific CXCR5^+^CD8^+^ T cells over time, while T cell activation genes were significantly downregulated in bulk Tet^–^CXCR5^+^CD8^+^ T cells (*ITM2A*, *KLRK1*) and in HIV-specific Tet^+^CXCR5^–^CD8^+^ T cells (e.g., *CD247*, *CD48*). HIV-specific Tet^+^CXCR5^+^CD8^+^ T cells showed even lower mRNA levels for genes associated with T cell exhaustion at the late time point compared with their CXCR5^–^ counterpart (e.g., *TOX*). We did not detect any DEGs associated with CD8^+^ T cell effector function in HIV-specific Tet^+^CXCR5^+^CD8^+^ T cells, while bulk Tet^–^CXCR5^+^CD8^+^ T cells increased some effector functions, and HIV-specific Tet^+^CXCR5^–^CD8^+^ T cells showed downregulation of DEGs related to effector function (e.g., *GZMB*) after 48 weeks of ART. In line with the CXCR5^+^ phenotype described earlier, bulk Tet^–^CXCR5^+^CD8^+^ T cells exhibited higher expression of genes encoding inflammatory chemokines and cytokines at week 48, with no change detected in the other 2 sorted CD8^+^ T cell populations. HIV-specific Tet^+^CXCR5^–^CD8^+^ T cells enhanced their tissue-homing phenotype over prolonged ART treatment (upregulation of *CCR7* and *CD69*, downregulation of *LSP1* and *S1PR1*), with few tissue homing and egress DEGs detected in the general CXCR5^+^ T cells over time. Yet, HIV-specific Tet^+^CXCR5^+^CD8^+^ T cells also upregulated the bone marrow–homing receptor gene *CXCR4*. Gene set enrichment analyses (Figure 7D) revealed that pathways associated with viral responses were upregulated in HIV-specific Tet^+^CXCR5^+^CD8^+^ T cells, downregulated in HIV-specific CXCR5^–^CD8^+^ T cells, and not differentially regulated in Tet^–^CXCR5^+^CD8^+^ T cells over the treatment course (Supplemental Figure 6C).

Taken together, our longitudinal flow cytometric analyses of circulating CXCR5^+^CD8^+^ T cells showed a preservation of HIV-specific effector functions by early ART over 48 weeks of suppressive therapy. Longitudinal transcriptomic analyses revealed an increased viral response signature at week 48, hinting toward consistent virus contact in CXCR5^+^ HIV-specific Tet^+^CD8^+^ T cells, but not in CXCR5^–^ HIV-specific Tet^+^CD8^+^ T cells.

### CXCR5^+^CD8^+^ T cells of individuals treated during acute infection proliferate and upregulate HIV-specific effector functions after in vitro stimulation and PD1 blockade.

**Figure 8:**
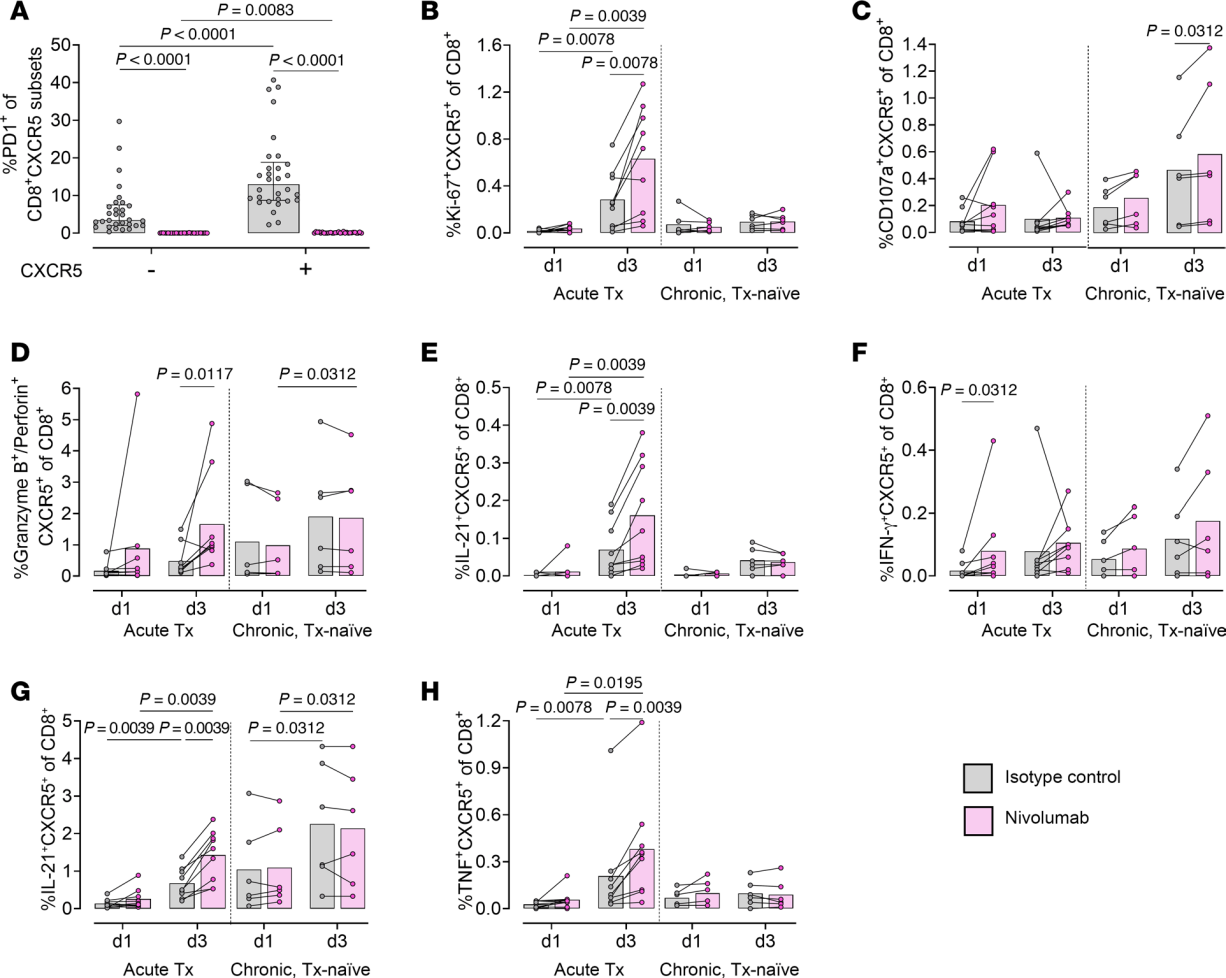
CXCR5^+^CD8^+^ T cells of individuals treated during acute infection proliferate and upregulate HIV-specific effector functions after in vitro stimulation and PD1 blockade. PBMCs were stimulated with HIV gag peptide pool for 20 hours (day 1) or 68 hours (day 3) with PD1 blockade (nivolumab; pink) or isotype control (gray). (**A**) PD1 expression on CXCR5^–^CD8^+^ and CXCR5^+^CD8^+^ T cell subsets (day 1 [d1] and d3 merged). (**B**–**H**) Frequency of proliferation and effector function markers coexpressed with CXCR5 on CD8^+^ T cells, as measured by intracellular cytokine staining assays. Left: Individuals treated during acute infection (12 weeks on ART; *n* = 9). Right: Individuals with chronic infection (Tx-naive; *n* = 3 LTNP and *n* = 3 viremic). The following markers are depicted: Ki-67 (**B**), CD107a (**C**), granzyme B/perforin (**D**), IL-2 (**E**), IFN-γ (**F**), IL-21 (**G**), and TNF (**H**). Mean values are shown and *P* values were calculated with Wilcoxon’s matched-pairs signed-rank test.

Next, we investigated whether PD1 blockade with nivolumab enhances anti-HIV effector functions of CXCR5^+^CD8^+^ T cells, which not only express high levels of PD1 but also of CD127, enabling them to respond to proliferation signals (Figure 6F and Supplemental Figure 5H). As expected, CXCR5^+^CD8^+^ T cells expressed significantly more PD1 than CXCR5^–^CD8^+^ T cells, and nivolumab abrogated PD1 staining (Figure 8A). Nivolumab led to an increased proliferation of CXCR5^+^CD8^+^ T cells as measured by Ki-67 (Figure 8B), which was accompanied by increased HIV-specific cytokine production (Figure 8, C–H), including the cytotoxic effector proteins granzyme B and perforin, an effect almost exclusively observed in individuals treated during acute infection. Notably, these nivolumab-dependent effects were nearly absent in CXCR5^–^CD8^+^ T cells (Supplemental Figure 7).

In summary, these in vitro experiments on human PBMCs extend the findings of others in small animal and SIV models (45, 84, 85) that CXCR5^+^CD8^+^, but not CXCR5^–^CD8^+^ T cells, respond to PD1 blockade with proliferation and upregulation of virus-specific effector functions. Importantly, we demonstrate that the effects of checkpoint inhibition are limited to individuals treated during acute HIV infection.

## Discussion

The viral reservoir persisting in lymphoid tissues (23, 25, 86), even in the face of ART (2–8) and natural immunity (5, 6, 8), remains a substantial obstacle to HIV eradication (10, 87). Functional CD8^+^ T cells play a pivotal role in controlling HIV/SIV (11–20), but their access to BCFs, a relevant reservoir site, depends on CXCR5 expression (6, 32, 34, 61). Early ART, while not preventing the establishment of an HIV reservoir, is associated with a swifter decline of the infected cell pool (46–48), preserved immune effector functions (88), and rare cases of posttreatment control (54, 55, 89). These observations identify individuals treated immediately during primary infection as promising candidates for functional HIV cure strategies (90, 91).

To explore the therapeutic potential of CXCR5^+^CD8^+^ T cells for HIV cure, we studied early ART’s impact on their functionality and association with the viral reservoir in individuals treated during primary HIV infection. Early ART not only preserved HIV-specific effector functions in CXCR5^+^CD8^+^ T cells, but also expanded a subset of CXCR5^+^CD8^+^ T cells coexpressing PD1 and CD127, enabling proliferation upon PD1 blockade. In line with this, only CXCR5^+^CD8^+^ T cells from individuals with early ART initiation responded to anti-PD1 treatment. Additionally, we demonstrate that numbers and HIV-specific effector functions of CXCR5^+^CD8^+^ T cells correlated with the viral reservoir decline. These findings highlight their potential in functional cure strategies, as CXCR5^+^CD8^+^ T cells have access to BCFs (33, 35, 60) where they could be exploited to eliminate the residual viral reservoir after reactivation strategies (15, 92–94), especially in the group of individuals treated during acute infection.

Seminal studies show that elite controllers have higher frequencies of follicle-homing HIV-specific CD8^+^ T cells in lymph nodes (8) and cytolytic effector functions of those cells (95), underlining the importance of functional CXCR5^+^CD8^+^ T cells in controlling HIV. Combining flow cytometric and transcriptomic analyses of low-abundance circulatory HIV-specific CXCR5^+^CD8^+^ T cells enabled us to dissect their functional memory phenotype. We could confirm the distinct functional and transcriptional signatures of CXCR5^+^CD8^+^ T cells (8, 35, 58, 71, 79, 96–98), with expression of the key T follicular cell transcription factors *ID3* and *RUNX3* that guide the transition to memory follicular T cells featuring a low activation, low exhaustion, and low cytotoxic phenotype. Circulating CXCR5^+^CD8^+^ T cells were largely CD69^–^ and CD127^+^, demonstrating lower activation and a capacity to receive IL-7–mediated homeostatic proliferation and survival signals (35, 99). This is noteworthy, as a recent study in a nonhuman primate SIV model demonstrated that posttreatment control after early treatment initiation was associated with the development of long-term memory virus-specific CD8^+^ T cells (55).

Interestingly, although CXCR5^+^CD8^+^ T cells showed decreased gene expression of cytolytic molecules independently of HIV specificity, they exhibited antiviral effector capacity upon in vitro stimulation. This effect was not limited to cytotoxicity but included helper/regulatory effector (IL-10, IL-21) functions. Our observations support the hypothesis that CXCR5^+^CD8^+^ T cells exist in a memory-like, less exhausted state (35), but can proliferate and enhance effector functions upon antigen-triggered activation (34, 45, 61), a key profile in controlling chronic viral infections like HIV (100). In line with this, we observed a downregulation of innate IFN-stimulated genes alongside upregulation of TLRs and inflammatory cytokines and chemokines in CXCR5^+^CD8^+^ compared with CXCR5^–^CD8^+^ T cells (77). TLRs, expressed by activated antigen-specific CD8^+^ T cells (71, 101, 102), can provide costimulatory signals resulting in lower antigen thresholds, promoting IFN-γ production and inhibiting HIV replication (102, 103). Despite these phenotypic and functional differences, in vitro control of autologous virus replication was comparable between CXCR5^+^CD8^+^ and CXCR5^–^CD8^+^ T cells.

One limitation of our study is the lack of matched tissue-resident CXCR5^+^CD8^+^ T cells, preventing us from determining how our observations in circulation translate to the events taking place in lymphoid tissue or whether circulating CXCR5^+^CD8^+^ T cells indeed home to BCFs (38, 60). However, we found a marked downregulation of tissue-homing genes and genes associated with avoiding tissue exit cues (*S1PR5*, *SELL*, and *CD69*) (66) alongside enrichment of locomotion pathway genes in circulatory CXCR5^+^CD8^+^ T cells. These transcriptional signatures, together with expression of CXCR5, suggest recent tissue egress and potential recirculation into lymphoid tissue (104). The lack of tissue-resident memory T cell gene enrichment and low *RUNX3* expression, attenuating tissue residency genes, further aligns with a circulatory phenotype (52, 53). Additionally, CXCR5^+^ HIV-specific CD8^+^ T cells exhibited increased IFN response to virus and T cell activation, with simultaneously decreased T cell exhaustion under prolonged ART, not observed in their CXCR5^–^ counterparts. Together with the observed sustained PD1 expression on CXCR5^+^CD8^+^ T cells, this could be indicative of a sustained antigen stimulus, only present at reservoir sites during ART (37, 98). Interestingly, these cells also enhanced their tissue-homing phenotype over prolonged ART (upregulation of *CCR7* and *CD69*, downregulation of *LSP1* and *S1PR1*), indicating movement toward reservoir sites like lymphoid tissue. As a proof of concept, CAR T cells genetically modified to coexpress an SIV-specific CAR and CXCR5 not only homed to extrafollicular and follicular lymph node regions, but also colocated with SIV RNA^+^ cells within lymphoid follicles after peripheral infusion in the nonhuman primate SIV model (105, 106).

Our findings of a correlation between functional HIV-specific CXCR5^+^CD8^+^ T cells and levels of intact proviral HIV DNA after ART initiation align with SIV model evidence suggesting CXCR5^+^CD8^+^ T cells may control viral replication (1, 36, 71), especially within lymphoid tissue (6, 8, 36, 87, 106–109).

Notably, CXCR5^+^CD8^+^ T cells sampled from individuals naturally controlling HIV were detectable at high frequencies and displayed cytotoxic characteristics like those from individuals treated during acute infection in our experiments. Findings on immune correlates in people naturally controlling HIV are of particular interest, as they exhibit a hallmark of HIV “functional cure”: a long-term drug-free viral remission. Suppression of viral replication by lymphoid CD8^+^ T cells in elite controllers, even without detectable cytolytic activity, suggests that also noncytolytic CD8^+^ T cell functions are relevant for HIV control in lymphoid tissues (8). In our experiments, HIV stimulation elicited noncytolytic cytokine responses (IL-10, IL-21), and increased transcription of noncytolytic effector molecules (e.g., chemokines and S100 protein) and innate IFN-γ–response signatures in CXCR5^+^CD8^+^ T cells, indicating an additional noncytotoxic immunoregulatory function of these cells.

In our experiments, approximately 20% of circulating CXCR5^+^CD8^+^ T cells coexpressed PD1 and CD127 independently of HIV infection, a phenotype that was absent in their CXCR5^–^ counterparts. CD127 supports homeostatic T cell proliferation (99, 110), which may explain why, in a chronic LCMV model, the proliferative burst after PD1 blockade came from a CXCR5^+^CD8^+^ T cell subset (45). Our findings that PD1 inhibition increased HIV-specific effector functions of CXCR5^+^CD8^+^, but not CXCR5^–^CD8^+^ T cells after in vitro stimulation, are in line with this observation (45) and extend the findings from the nonhuman primate SIV model (84, 111, 112) to humans. Here, PD1 blockade enhanced and sustained the function of vaccine-induced CD8^+^ T cells and decreased viral reservoirs in lymphoid tissue (111). Furthermore, PD1 blockade expanded polyfunctional CXCR5^+^CD8^+^ T cells after ART interruption, improving control of re-emerging viremia (84). In summary, these findings from animal models, combined with in vitro findings by us and others (113, 114) as well as initial results of clinical trials (43, 115), call for further evaluation of this approach in future (pre)clinical studies aimed to increase HIV-specific effector functions of (CXCR5^+^) CD8^+^ T cells (15, 44, 116, 117). Suspected immune-related adverse events of PD1 blockade in otherwise healthy individuals, however, warrants careful participant selection for clinical trials (118). Notably, in our experiments, the PD1-blockade-induced increase in HIV-specific effector functions was limited to the group of individuals treated during acute infection and not observed in individuals with chronic infection.

In conclusion, our findings underscore the importance of early diagnosis and treatment for achieving functional cure of HIV; individuals treated during early infection exhibit a rapid reservoir decline, as demonstrated herein and by others (46–48), preserved immune effector functions (55, 88), and a unique responsiveness to PD1 blockade, positioning them as prime candidates for such interventions.

## Methods

Detailed information on reagents, devices, and antibodies is provided in Supplemental Tables 3–7.

### Sex as a biological variable.

In Germany, approximately 80% of people with newly diagnosed HIV are male. Therefore, predominantly male participants were recruited (Supplemental Table 8). The number of female participants did not allow separate analysis.

### Study participants.

Three clinical groups of people LWH were included in this study (Figure 1 and Supplemental Figure 1): (a) Individuals diagnosed and treated during acute HIV infection (Fiebig I–IV), enrolled into the Treatment of Primary HIV-1 Infection (TopHIV) cohort of the German Center for Infection Research (DZIF) (*n* = 37). This cohort includes participants receiving ART following the first study blood draw (TopHIVFUTURE; *n* = 27) and individuals at least 6 weeks on ART at the first blood drawl (TopHIVPAST; *n* = 10). Furthermore, we included (b) individuals with chronic HIV infection before and after ART initiation (chronic; *n* = 10) as well as (c) individuals naturally controlling HIV, defined as plasma viral RNA concentration of less than 2,000 copies/mL in the absence of ART (“LTNPs”; *n* = 20). As controls, people LWOH (*n* = 10) were included in the flow cytometry experiments. To analyze the effect of early ART initiation, participants of TopHIVFUTURE after 48 weeks of treatment and TopHIVPAST cohort were combined into “acute Tx, treated during acute infection” (*n* = 37; Supplemental Figure 1). A further 8 ART-naive people chronically LWH were included for viral inhibition assay (*n* = 3) or transcriptomic analysis (*n* = 5). Tonsil tissue was obtained from patients undergoing routine tonsillectomy around KwaZulu-Natal, South Africa. Detailed participant characteristics are provided in Supplemental Table 1.

### Sample processing.

PBMCs were isolated by Histopaque density-gradient centrifugation, resuspended in precooled cryo media, and transferred to liquid nitrogen. Tonsil tissue samples were fixed in paraformaldehyde (16–24 hours), embedded in paraffin wax, and stored at room temperature.

### Antibody and peptide-MHC Tet staining for flow cytometry.

To characterize CXCR5^+^CD8^+^ T cells by flow cytometry and to sort T cells for low-input RNA sequencing, thawed PBMCs were either stained directly or rested in R10 medium overnight (37°C, 5% CO_2_). Cells were stained with APC-conjugated peptide-MHC-I (pMHCI) tetramers for 30 minutes at room temperature. The following APC-conjugated tetramers were used: HLA-A*02:01-SLYNTVATL (SL9, gag 77-85; provided by David A. Price, University Hospital of Wales, Cardiff), HLA-B*07:02-RPQVPLRPM (RM9; nef 71–79), and HLA-B*40:02-KEKGGLEGL (KL9, nef 92–100). Biotinylated pMHCI monomers were conjugated with APC-conjugated streptavidin to form tetrameric pMHCI complexes. After PBS washes, cells were stained for viability and incubated with fluorochrome-conjugated antibodies (20 minutes). Cells were washed, resuspended in FACS buffer for cell sorting, or fixed and washed. TLR8 staining included additional permeabilization, intracellular antibody staining, washing, and resuspension in PBS. Rainbow beads were used to ensure interexperimental consistency. Data were analyzed with FlowJo software, version 10 (Tree Star). The gating strategy is shown in Supplemental Figure 8. Positive gates were selected using fluorescence-minus-one (FMO) controls. Memory subsets and combined PD1 and CD127 analyses were obtained using Boolean gates.

### Preselection of HIV-specific CD8^+^ T cell populations for transcriptomic analyses.

To identify participants for transcriptomic analyses of HIV-specific T cells, we screened 25 study participants during acute infection for HIV gag and nef responses using IFN-γ ELISpot assays, as described previously (119, 120). Briefly, 100,000 thawed PBMCs per well were added to 96-well plates coated overnight with anti–human IFN-γ monoclonal antibody and containing overlapping peptides. SEB (staphylococcal enterotoxin B) and FEC (influenza virus, EBV, CMV) peptide pools were used as positive controls, while media-only wells served as negative controls. After overnight incubation (37°C, 5% CO_2_) and subsequent washes with PBS, the biotinylated IFN-γ detection antibody was added. Signal generation was performed using an AP Conjugate Substrate Kit. Spots were counted using an automated ELISpot plate reader and the number of spot-forming cells (SFC) was calculated by subtracting the mean of the negative controls. Positive reactions were defined as 3 or more times the mean SFC of the negative controls and greater than 50 SFC/10^6^ PBMCs.

Furthermore, 20 individuals with the A*02:01 HLA allele were screened for HIV gag SL9 (72) recognition via Tet staining as described above. Using the ELISpot results combined with in silico prediction tools (121) and consecutive confirmation by Tet staining, we identified 1 individual recognizing KL9 (73, 74) and 3 individuals recognizing RM9 (75). For chronic infection, we identified 1 individual recognizing RM9 and 5 individuals recognizing SL9 (72) by Tet staining.

### Multiplex immunofluorescence microscopy.

Multiplex immunofluorescence microscopy was performed using the Opal 4-Color Manual IHC Kit according to the manufacturer’s instructions. Briefly, tonsil sections (4 μm) were deparaffinized and stained with unlabeled primary antibodies. Opal fluorophores and imaging filters used were as follows: Opal520 with qFITC for CXCR5, Opal570 with qTexa for CD8, and Opal690 with qCy5 for HIV p24. DAPI was used as the nuclear counterstain. Images were acquired at ×40 magnification using a Zeiss Axio Z2 microscope.

### Low-input RNA sequencing.

HIV-specific CD8^+^ T cells from individuals identified by ELISpot were selected via Tet staining (see above) for low-input RNA sequencing. For each of the 4 cell populations (CXCR5^+^Tet^+^, CXCR5^+^Tet^–^, CXCR5^–^Tet^+^, and CXCR5^–^Tet^–^), 1–3 wells containing up to 50 live cells per well were sorted sequentially. Supplemental Figure 8C describes the gating strategy. RNA isolation and library preparation was performed using Smart-seq2 (122). Briefly, cells were sorted directly into a 96-well plate containing 5 μL lysis buffer. Full-length RNA was reverse transcribed by SuperScript II Reverse Transcriptase using oligo(dT) primers and a template switching primer, both carrying an additional sequence to prime the subsequent amplification PCR (0.2 μM universal primer; KAPA HiFi HotStart ReadyMix). Libraries were prepared with a Nextera XT DNA Library Preparation Kit and Nextera XT Index Kit v2 Set according to the manufacturer’s instructions, but in a quarter of the volume. PCR products and libraries were isolated using Agencourt AMPure XP beads. DNA was quantified by Qubit and size distribution was assessed using the Agilent High Sensitivity DNA Kit. Sequencing (100-bp paired-end) of the libraries was performed on a HiSeq or NovaSeq Illumina instrument with an average sequence depth of 1–4 × 10^6^ reads per cell. Quality of raw sequencing reads was assessed using FastQC (https://www.bioinformatics.babraham.ac.uk/projects/fastqc/) and MultiQC (123). Sequence reads were trimmed to remove adapter sequences and nucleotides with poor quality (Phred score < 3) using cutadapt v4 (https://doi.org/10.14806/ej.17.1.200). The trimmed reads were mapped to the *Homo* *sapiens* reference genome GRCh38/hg19 using the STAR aligner in paired-end mode (124). Unique gene hit counts were calculated by using feature Counts from the Subread package (125). Only unique reads within exon regions were counted. Genes were kept only when they were protein coding and nonmitochondrial genes as well as their expression was detected in at least 50% of samples in either of the comparison group. The resulting raw gene counts table served as input for DESeq2 (126) to perform multiple pairwise comparisons of gene expression between immune cell subsets. The poscounts option in Deseq2 was used to account for the low input samples. *P* values were adjusted for multiple testing using the method by Benjamini and Hochberg (BH). Shrinkage estimator ashr was used to shrink log_2_(fold change) (127). DEGs between individual comparisons were defined by a false discovery rate–corrected *P* value of less than 0.05 and absolute log_2_(fold change) of 1 or greater. The R package clusterProfiler (128) was used to explore the biological pathways of DEGs using GO enrichment analysis. Top listed pathways were defined with a corrected *P* value of less than 0.05. GSEA was performed using the R package fgsea (129).

### Intracellular cytokine staining after antigenic stimulation.

Thawed PBMCs were either left unstimulated or stimulated with HIV SF2 overlapping peptide pools spanning gag proteins (15–20 aa long, overlap of 5–10 aa) or SEB (positive control) in the presence of anti-CD107a. After 1 hour, cells were supplemented with anti-CD28, anti-CD49d, Brefeldin A, and GolgiPlug protein transport inhibitor and incubated for 14 hours (37°C, 5% CO_2_). Cells were washed and stained for viability and cell surface markers as described above. For intracellular staining, cells were fixed and permeabilized, incubated with 20% goat serum for Fc receptor blockade, and stained with antibodies against intracellular antigens for 20 minutes. After washing, cells were resuspended in PBS and acquired. Rainbow beads were used to ensure interexperimental consistency. The data were analyzed with FlowJo software, version 10 (Tree Star). The gating strategy is shown in Supplemental Figure 9, A and B. Positive gates were selected using FMO controls. Cell type frequencies were calculated by background subtraction except granzyme B and perforin. For these, the unstimulated values were used, as the reconstitution of intracellular perforin following degranulation has been reported to first require cellular proliferation (130–132). To define cytotoxicity (granzyme B^+^perforin^+^) and cytokine production as a function of PD1 and CD127, Boolean gates were used.

### Quantification of cell-associated HIV DNA.

Cell-associated HIV-1 DNA was quantified using a modified protocol of the intact proviral DNA assay (IPDA) (83). Briefly, CD4^+^ T cells were isolated from cryopreserved PBMC samples by immunomagnetic negative selection using the EasySep Human CD4 T Cell Isolation Kit. DNA was isolated using DNAzol and quantified using a Quantus Fluorometer. Genome equivalents were quantified in a 1:50 dilution by digital PCR using 2 targets in the *RPP30* gene to account for DNA shearing. DNA corresponding to 200,000–300,000 genome equivalents was used as input for the IPDA on a Qiacuity One 5-plex digital PCR instrument with 26K Nanoplates using the Qiacuity Probe PCR kit. IPDA primers and probes for the Psi and Env targets were optimized to increase coverage of nonsubtype B samples (Supplemental Table 7). HIV-uninfected PBMCs and J-Lat clone 8.6 cells (NIH AIDS Reagent Program) served as negative and positive controls, respectively. Partitions with positive signals for Env and Psi were considered as potentially “intact” proviruses. Results are expressed as HIV-1 copy numbers per million cell equivalents. Participants with at least 50,000 genome equivalents were included in the analysis.

### Viral inhibition assay.

To assess the capacity of CXCR5^+^CD8^+^ T cells to control viral replication in vitro, we performed a coculture assay with stimulated CD4^+^ and CXCR5^+^CD8^+^ or CXCR5^–^CD8^+^ T cells. Due to the low frequency of circulating CXCR5^+^CD8^+^ T cells, this assay was performed with samples from treatment-naive individuals during chronic infection (PBMCs from 2 time points were pooled) (Supplemental Tables 1 and 2). CD4^+^ T cell were separated by negative selection using the EasySep Human CD4 T Cell Isolation Kit following the manufacturer’s protocol. Isolated CD4^+^ T cells were suspended at 1 × 10^6^ cells/mL in R10-50 and stimulated for 4 days (37°C, 5% CO_2_) with 1 μg/mL PHA as described elsewhere (133). On the day of assay setup, CXCR5^+^CD8^+^ and CXCR5^–^CD8^+^ T cells were separated by FACS; approximately 1 × 10^8^ (minimum 1.3 × 10^8^) freshly thawed PBMCs, matching the donors of the separated CD4^+^ T cells, were stained with antibodies as described above. For live/dead discrimination, propidium iodide was added directly before sorting. Stimulated CD4^+^ T cells were washed and cocultured with sorted CXCR5^+^CD8^+^ or CXCR5^–^CD8^+^ T cells (effector/target ratio 1:1) in 100 μL R10-50. The same quantity of CXCR5^+^CD8^+^ and CXCR5^–^CD8^+^ T cells were used. Given sufficient CXCR5^+^CD8^+^ T cells, experiments were run in duplicate on a 96-well plate. CD4^+^ T cells without CD8^+^ T cells were cultured in duplicate in R10-50 at the same cell number as in coculture. Cells were incubated for 15 days (37°C, 5% CO_2_) with 60 μL supernatant collected and replenished on days 3, 6, 9, 12, and 15 and analyzed for HIV p24 production using a quantitative sandwich ELISA following the manufacturer’s protocol. HIV p24 concentration was determined by interpolation from standard curve. Only wells with HIV p24 concentrations greater than 0 or double-negative wells were included.

### PD1 blockade assay.

The impact of PD1 blockade on CD8^+^ T cell function was analyzed in an intracellular cytokine staining flow cytometric assay (see above) with and without PD1 blockade (nivolumab). Thawed PBMCs were adjusted to 2 × 10^6^ cells/mL in R10 containing ART. Rested (2 hours) PBMCs were stimulated with either 0.5 μg/mL gag peptide pool, 1 μg/mL SEB (positive control), or left without stimulation. Simultaneously, cells were treated with nivolumab or IgG4 isotype control. Staining was performed 20 hours (day 1) and 68 hours (day 3) after stimulation. Data were analyzed with FlowJo software, version 10 (Tree Star) and gating was guided by FMO controls (gating strategy in Supplemental Figure 9C).

### Statistics.

Statistical analyses were conducted with GraphPad Prism software version 10.1.0. The Mann-Whitney test or Wilcoxon’s matched-pairs signed-rank test was used for 2-group comparison, while Kruskal-Wallis or Friedman’s test with Dunn’s correction for multiple comparisons was applied for comparisons of more than 2 groups. *P* values of less than 0.05 were considered significant. Correlations were performed with Spearman’s rank method. The data are expressed as median and interquartile range (IQR) unless specified otherwise. Statistical methods for transcriptomic analyses are described above.

### Study approval.

Blood samples of individuals treated during acute infection were collected in the TopHIV cohort (DZIF), coordinated by the translational platform HIV (TP-HIV), and approved by the Institutional Review Board of the University of Cologne, Germany (protocol 13-364). Blood samples of individuals with chronic HIV infection were collected in studies approved by the Institutional Review Board of the Ludwig-Maximilians-Universität, Munich, Germany (protocols 274-03,179-16, and 19-849). Tonsil tissue collection was approved by the University of Kwa-Zulu Natal (ref: BE061/13). All participants provided written informed consent at the time of enrollment.

### Data availability.

Low-input RNA sequencing data are available in the European Nucleotide Archive (accession code: PRJEB85160). Supporting Data Values file contains all graph data points. Raw data will be made available upon reasonable request to the corresponding author.

## Authors contributions

JR designed the study. SR performed the acquisition, analysis, and interpretation of the flow cytometric data supported by RS, RC, and JR. SR developed and performed the viral inhibition assay supported by JE, HN, RS, and JR. SR and VO identified the optimal epitopes for selection of HIV-specific CD8^+^ T cells. FSA conducted immunofluorescent staining of tonsil tissue. TV and MM quantified cell-associated HIV DNA. E Gruener developed, performed, and analyzed the PD1 blockade assay. KH, DW, and LR performed RNA sequencing experiments and analysis. IA performed and supported cell sorting. RZ performed HLA class I typing. E Gersbacher, JE, RP, NP, CDS, JRB, and US recruited participants for the TopHIV-cohort. JJV and MS managed the TopHIV-cohort and provided participant clinical data. RD provided samples. HNK and AL provided tonsil samples. SR, E Gruener, DW, JR, and KH wrote the manuscript, which was critically reviewed and edited by CG, MM, HNK, and AL. JR, KH, HNK, CG, RD, and MM acquired funding. The co–first authorship order reflects contribution. All authors read and approved the final manuscript.

## Supplementary Material

Supplemental data

Supplemental table 1

Supporting data values

## Figures and Tables

**Table 1:**
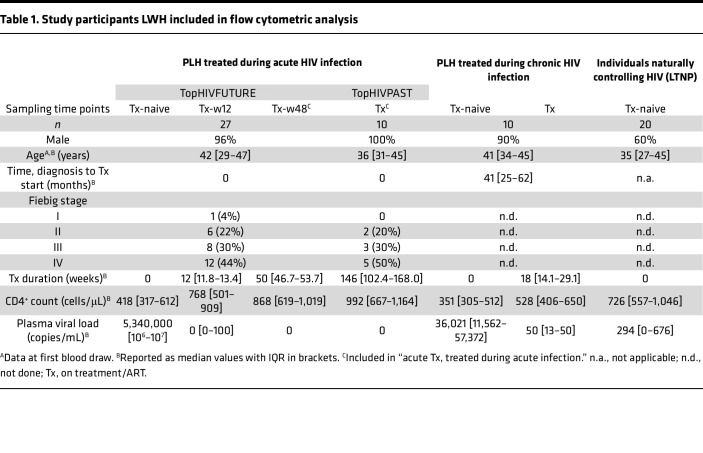
Study participants LWH included in flow cytometric analysis

## Version 1 04/08/2025

Electronic publication
