# Supplementary material for: Early treatment and PD1 inhibition enhance HIV-specific functionality of follicular CD8+ T cells
Source: JCI Insight. 2025 Apr 8;10(7):e180309. doi: 10.1172/jci.insight.180309 (PMC11981630; doi:10.1172/jci.insight.180309)
Supplement: Supplemental data [file jciinsight-10-180309-s035.pdf]

Supplemental Information

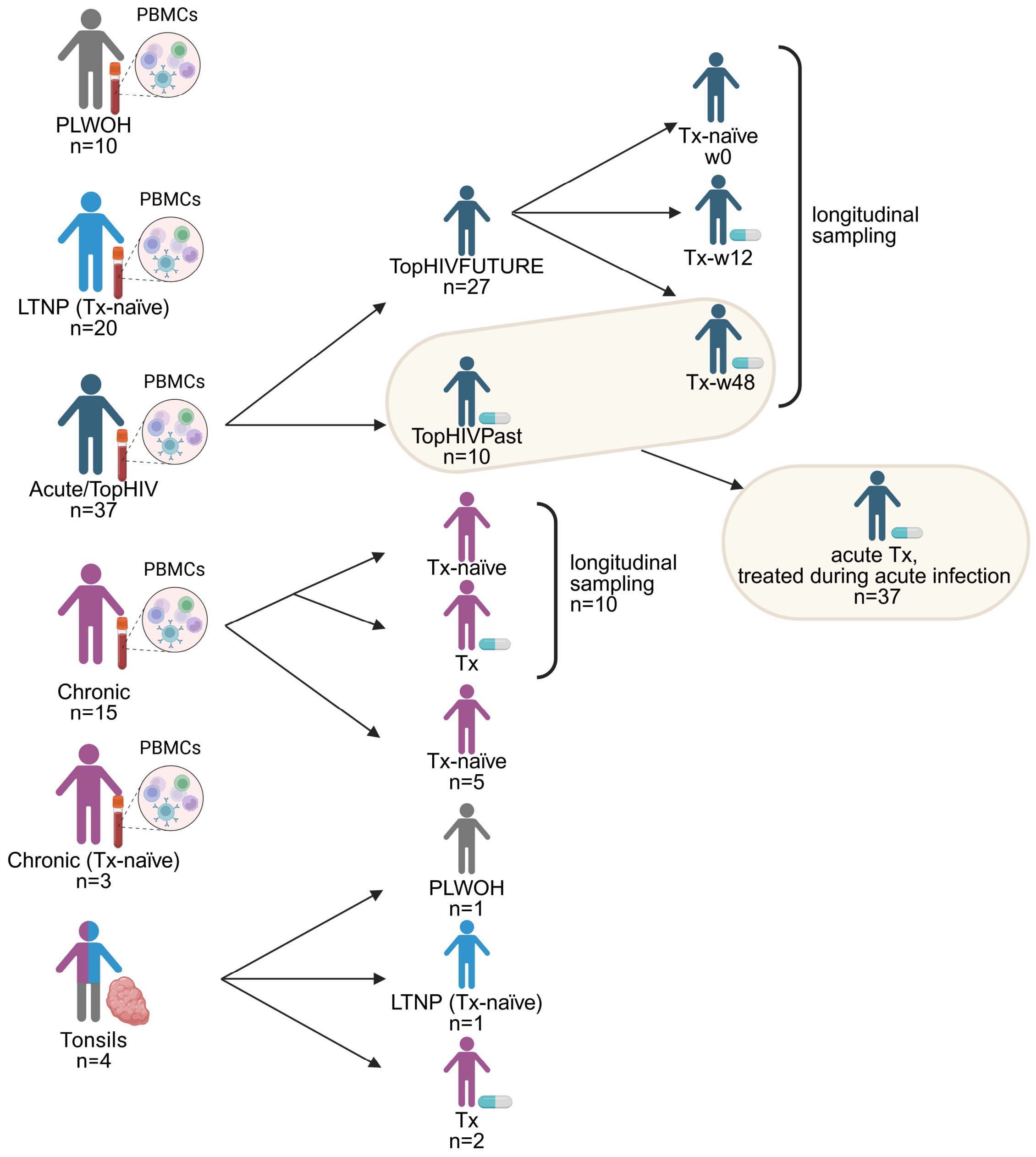

Supplemental Figure 1: Schematic representation of the cohort and sampling timepoints used for analyses. Created in BioRender. Immunity, I. (2025) https://BioRender.com/i33u513.

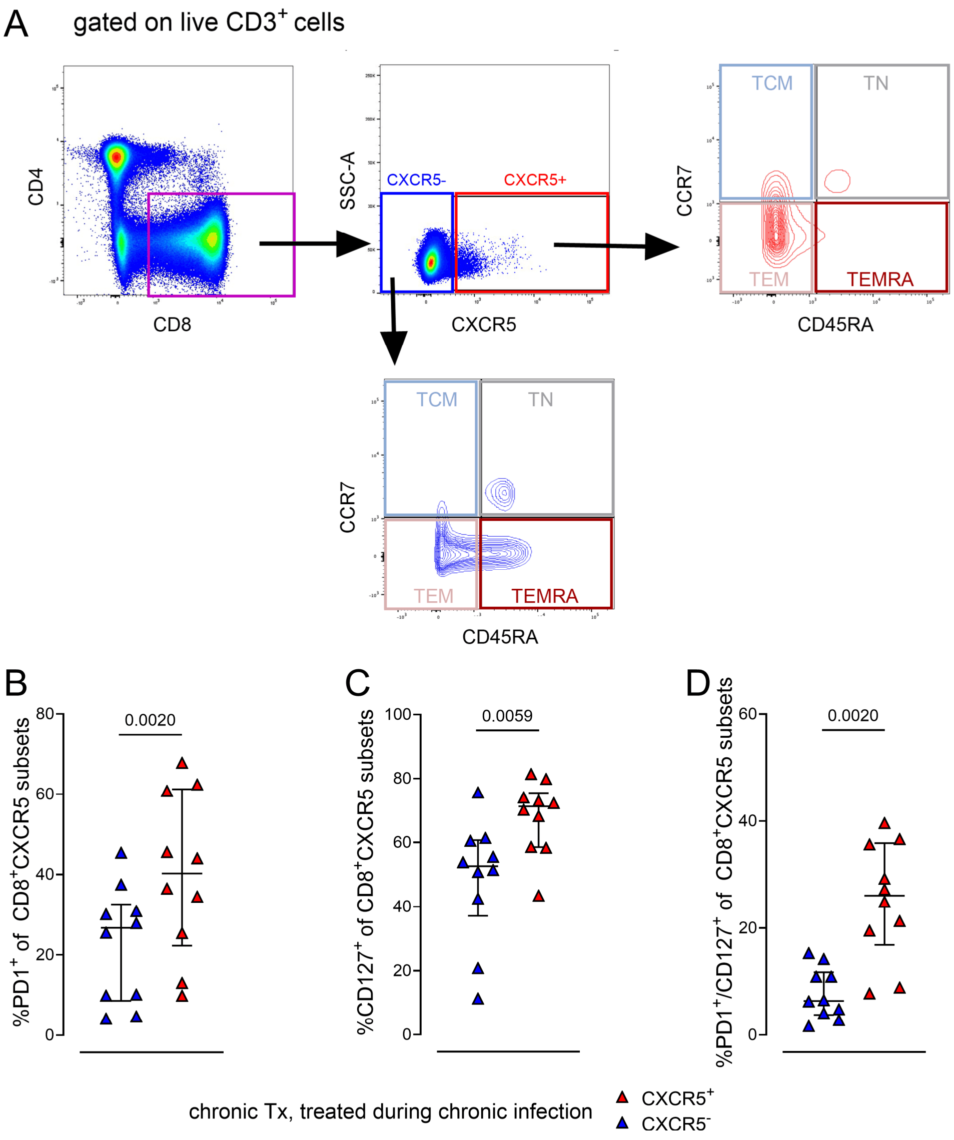

Supplemental Figure 2: Memory subset distribution in CXCR5+ vs CXCR5- CD8 T cells.
(A) Exemplary gating strategy for memory subset distribution in CXCR5+ vs CXCR5- CD8 T cells. TCM: central memory (CCR7+CD45RA-), TN: naïve (CCR7+CD45RA+), TEMRA: effector memory re-expressing CD45RA (CCR7-CD45RA+), TEM: effector memory (CCR7-CD45RA-). **(B-D)** Frequencies of CXCR5- (blue) and CXCR5+ (red) CD8 T cells expressing PD1 **(B)**, CD127 **(C)** and co-expressing PD1 and CD127 **(D)** in individuals diagnosed and treated during chronic infection (n=10). P-values by Wilcoxon matched-pairs signed rank test. Medians and IQRs are indicated.

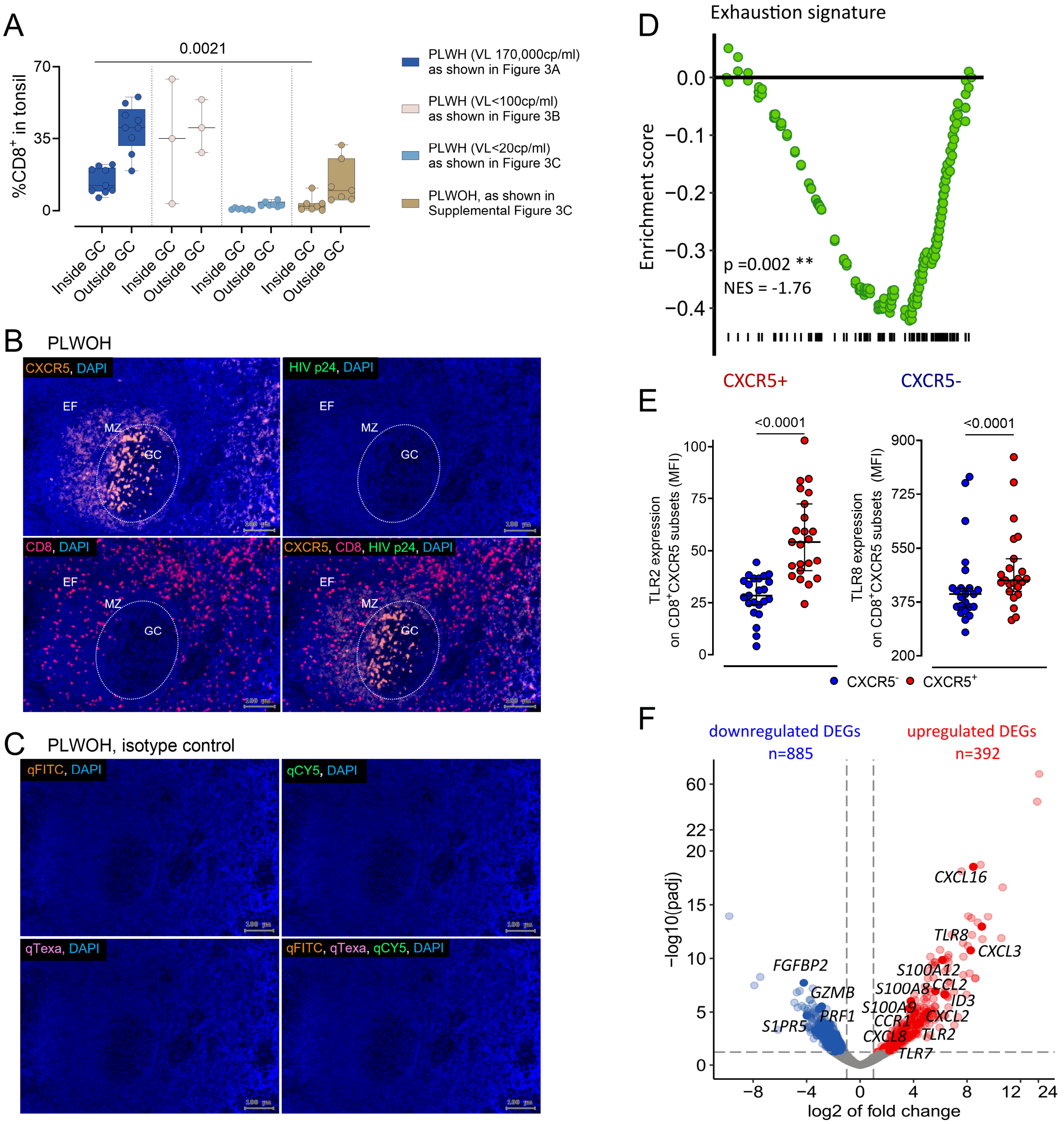

Supplemental Figure 3: Differing transcriptomic profile in CXCR5- and CXCR5+ CD8 T cells and confirmation of selected DEGs at a protein level.

(A) Quantification of CD8 T cells inside and outside the germinal center (GC) in tonsil stains from the same donors as shown in Figure 3, A-C and Supplemental Figure 3B. Percentage positivity is calculated with the following formula: %CD8 T cells=(area of positive region/total area)x100%. P-value was calculated with Mann-Whitney-test. **(B-C)** Immunofluorescence microscopy of tonsil tissue from a participant living without HIV: stained for CXCR5 (orange), CD8 (pink) and HIV p24 (green) **(B)** and mouse IgG isotype negative control which was used with each of the opal fluorophores **(C)** within germinal centers (GC), mantle zone (MZ) or extrafollicular (EF) regions. Spectral DAPI was used as a nuclear counterstain (blue). Imaging was performed with a Zeiss Axio Z2 microscope at 40x magnification and scale bars =100µm. **(D)** GSEA showing the enrichment of genes associated with exhaustion in CXCR5- but not CXCR5+ circulatory CD8 T cells. NES=normalised enrichment score. **(E)** Expression of selected proteins encoded by DEGs in people treated during acute HIV infection prior ART initiation (n=23) detected by flow cytometry in CXCR5+ and CXCR5- CD8 T cells. Median fluorescent intensity (MFI) of CXCR5 subsets and IQR are depicted. P-values were calculated with Wilcoxon matched-pairs signed rank test. **(F)** Volcano plot showing upregulated (red) and downregulated (blue) DEGs in CXCR5+ vs CXCR5- CD8 T cells in people living with HIV (n=13; max 4 weeks on ART). Cut-offs were set to adjusted p-value <0.05 and absolute log2 fold-change >1. Selected immune-related genes are highlighted.

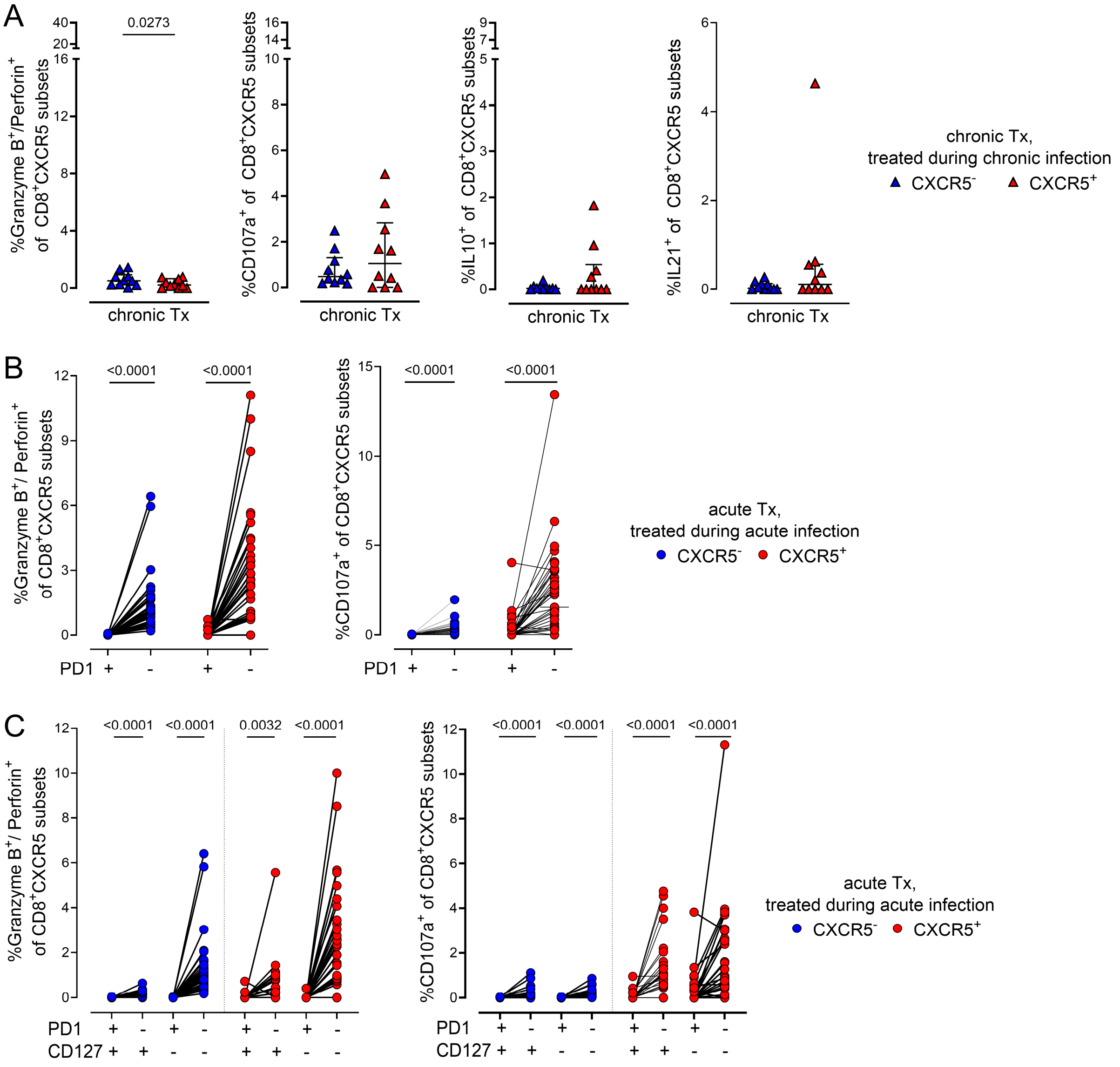

Supplemental Figure 4: Expression of several effector function markers following stimulation on CXCR5- vs CXCR5+ CD8 T cells.

(A) Frequency of CXCR5- (blue) and CXCR5+ (red) CD8 T cells co-expressing Granzyme B and Perforin ex-vivo, and CD107a, IL10, or IL21 after stimulation with HIV-gag in individuals diagnosed and treated during chronic infection (n=10). P-values by Wilcoxon matched-pairs signed rank test. Medians and IQRs are depicted. **(B and C)** Frequency of CXCR5- (blue) and CXCR5+ (red) CD8 T cells co-expressing Granzyme B and Perforin ex-vivo (left) or expressing CD107a (right) in response to overnight (O/N) stimulation with HIV-gag peptide pool in perspective to PD1 **(B)** or combined PD1 and CD127 **(C)** expression in individuals treated during acute infection (n=37; median time on treatment 52 weeks). P-values by Wilcoxon matched-pairs signed rank test.

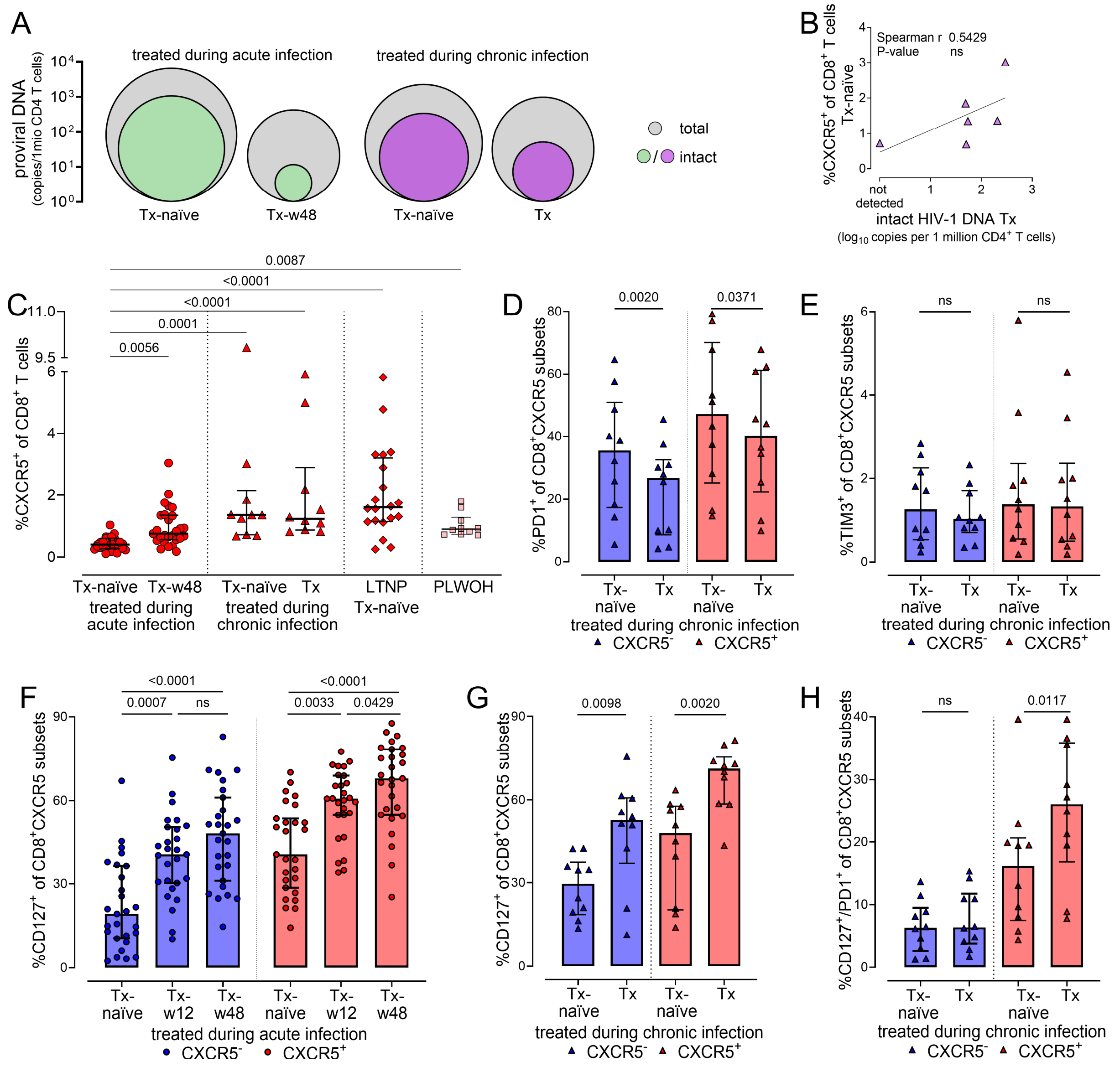

Supplemental Figure 5: Longitudinal dynamics of proviral HIV DNA and phenotypic markers in people treated during acute or chronic HIV infection.

(A) Median copies of intact (colored) or total (gray) proviral HIV DNA per one million CD4 T cells prior and during ART in individuals treated during acute (prior ART n=22; during ART n=25; green) and chronic (prior ART n=8; during ART n=6; purple) infection. **(B)** Association between the frequencies of CXCR5+ CD8 T cells prior ART and levels of intact proviral HIV-DNA in individuals treated during chronic infection (n=6; median time on ART 18 weeks). Spearman rank correlation was performed. **(C)** Frequencies of CXCR5+ CD8 T cells prior and during ART in individuals with acute (n=27; circles) and chronic (n=10; triangles) HIV infection, individuals naturally controlling HIV (n=20; diamonds) and people living without HIV (PLWOH; n=10; squares). P-values by Kruskal-Wallis-test with Dunn's correction for multiple comparisons. Medians and IQRs are depicted. **(D-H)** Effect of ART initiation on PD1 **(D)**, TIM3 **(E)**, CD127 **(F and G)** and CD127/PD1 **(H)** (co-)expression on CXCR5- (blue) and CXCR5+ (red) CD8 T cells in individuals treated during chronic (**D, E, G and H**; n=10; median time on ART 18 weeks) or acute (**F**; TopHIVFUTURE; n=27) infection. P-values by Wilcoxon matched-pairs signed rank test (comparison of two groups) or Friedman test with Dunn's correction for multiple comparisons (comparison of >2 groups). Medians and IQRs are displayed.

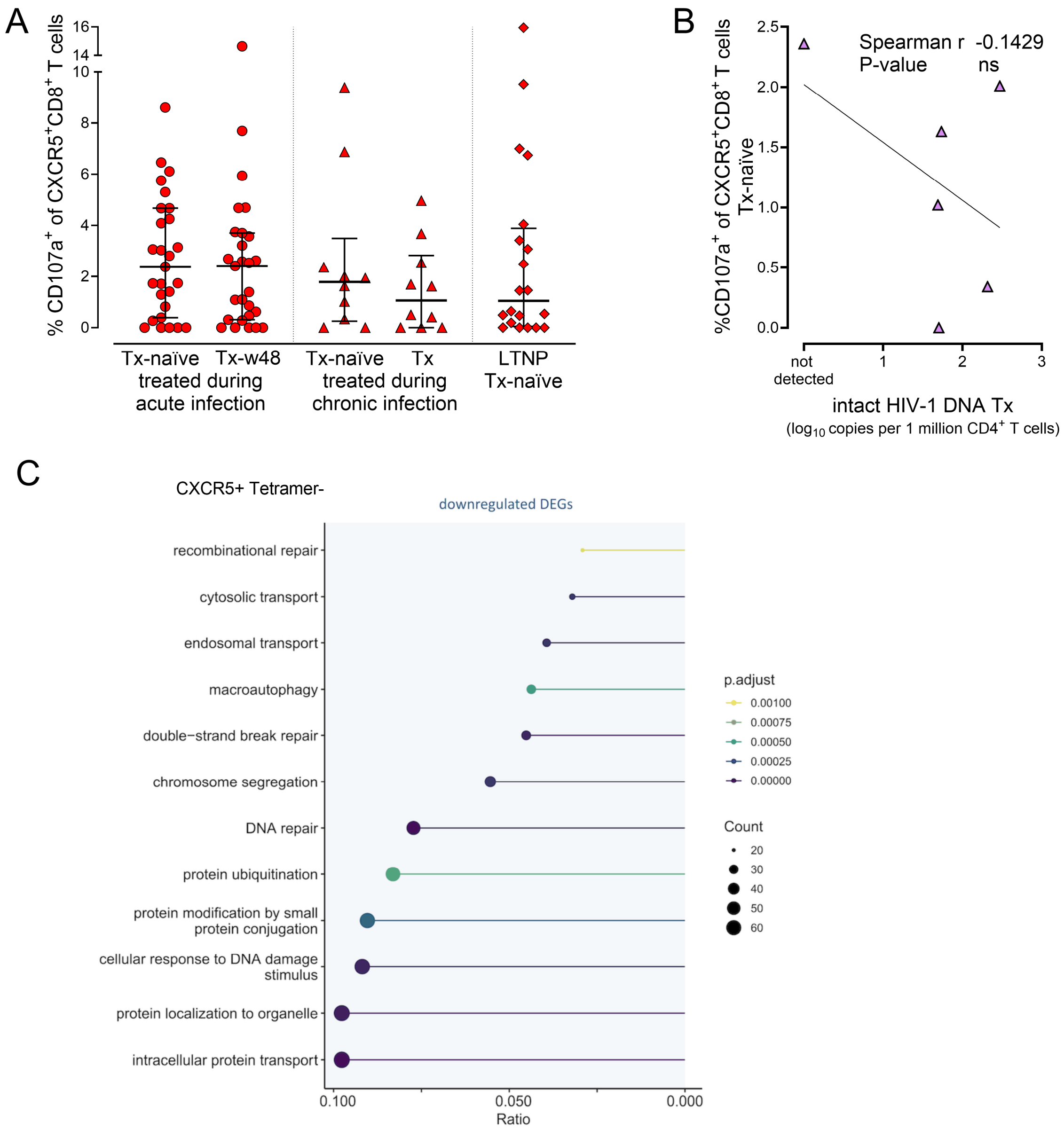

Supplemental Figure 6: Influence of ART initiation on CD107a expression and on downregulation of differentially expressed genes.

(A) Frequencies of CD107a+CXCR5+ CD8 T cells after O/N stimulation with HIV-gag peptide pool in individuals diagnosed and treated during acute (n=27; circles) or chronic infection (n=10; triangles) prior and during ART and in individuals naturally controlling HIV (LTNP; n=20; diamonds). Kruskal-Wallis-test corrected for multiple comparisons was performed. Medians and IQRs are displayed. **(B)** Association between HIV-specific CD107a-expressing CXCR5+ CD8 T cells responding to O/N stimulation with HIV-gag peptide pool at baseline and intact HIV DNA after median 18 weeks of ART in individuals treated during chronic infection (n=6). Spearman rank correlation was performed. **(C)** Enriched GO terms (biological process) for significantly differentially expressed genes that are downregulated in CXCR5+ CD8 T cells w4 vs w48 post ART initiation (BH adjusted p-values $<0.1*10^{-2}$). Length of the line represents the ratio of the number of observed differentially expressed genes (DEGs) per pathway and total number of DEGs, dot size represents the absolute observed number of DEGs per pathway. Color code is according to the adjusted p-value.

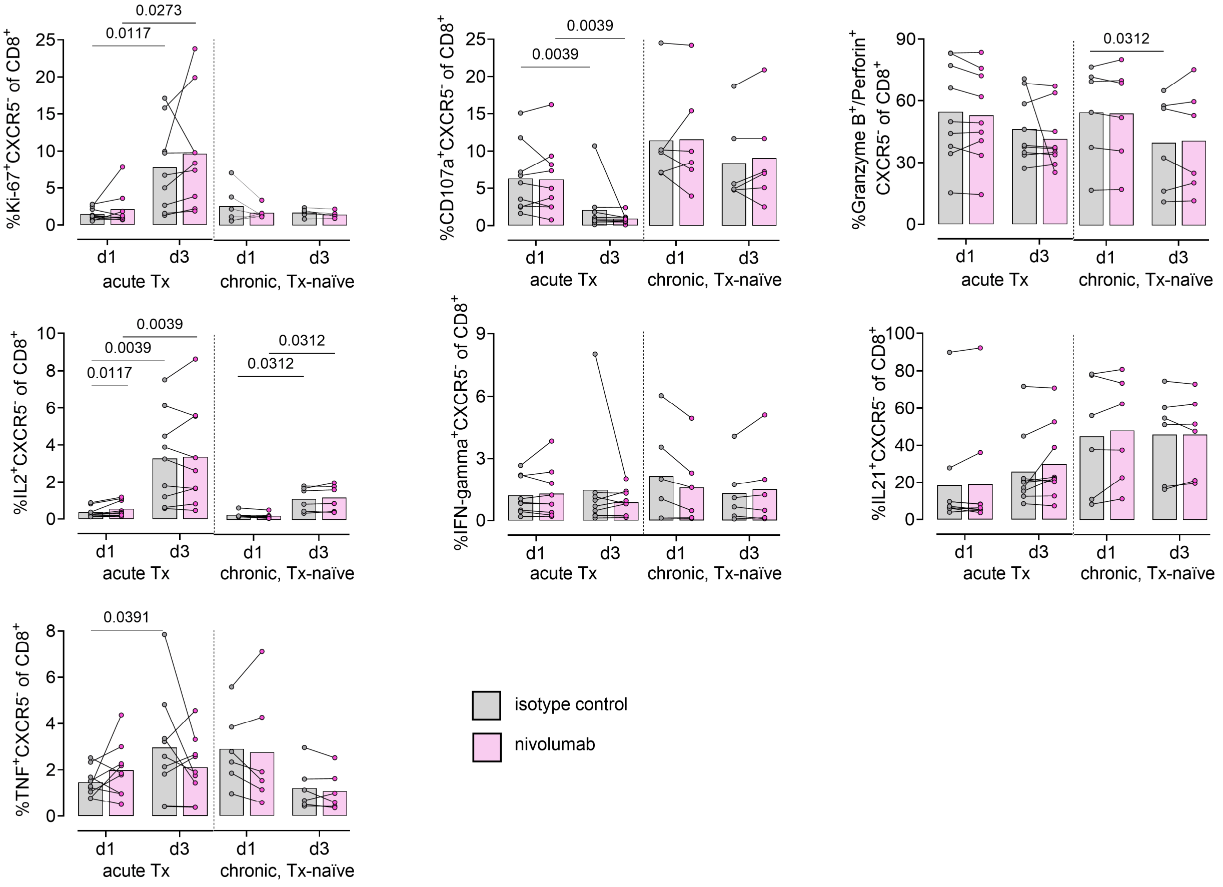

Supplemental Figure 7: Nearly absent influence of PD1-blockade on CXCR5- CD8 T cells.

PBMC were stimulated with HIV-gag peptide pool for 20h (d1) or 68h (d3) in presence of a PD1-inhibitor (nivolumab; pink) or isotype control (gray) as described for the corresponding Figure 8. Expression of markers for proliferation (Ki-67), cytotoxicity (CD107a, Granzyme B and Perforin), and non-cytotoxic effector functions (IL2, IFN-gamma, IL21, TNF) of CXCR5- CD8 T cells from individuals treated during acute infection (left; 12 weeks on ART; n=9) or living with chronic HIV infection (right; Tx-naïve; n=3 viremic and n=3 naturally controlling HIV). P-values are calculated with Wilcoxon matched-pairs signed rank test.

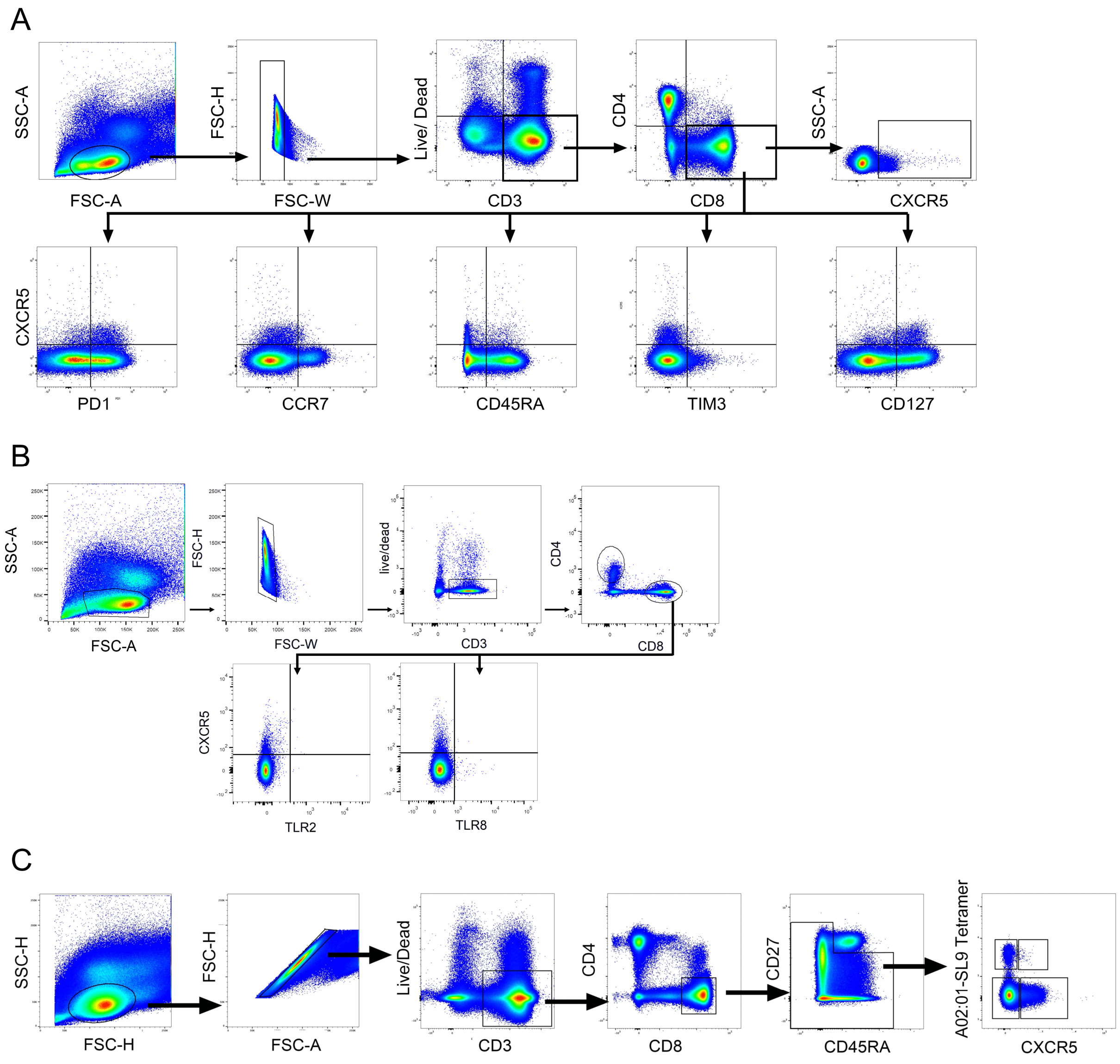

Supplemental Figure 8: Exemplary gating strategy for phenotypic markers.
(A) Extracellular markers detected by flow cytometry, **(B)** confirmation of the expression of selected DEGs by flow cytometry and **(C)** FACS gating strategy for tetramer sorting of HIV-specific CD8 T cells are shown.

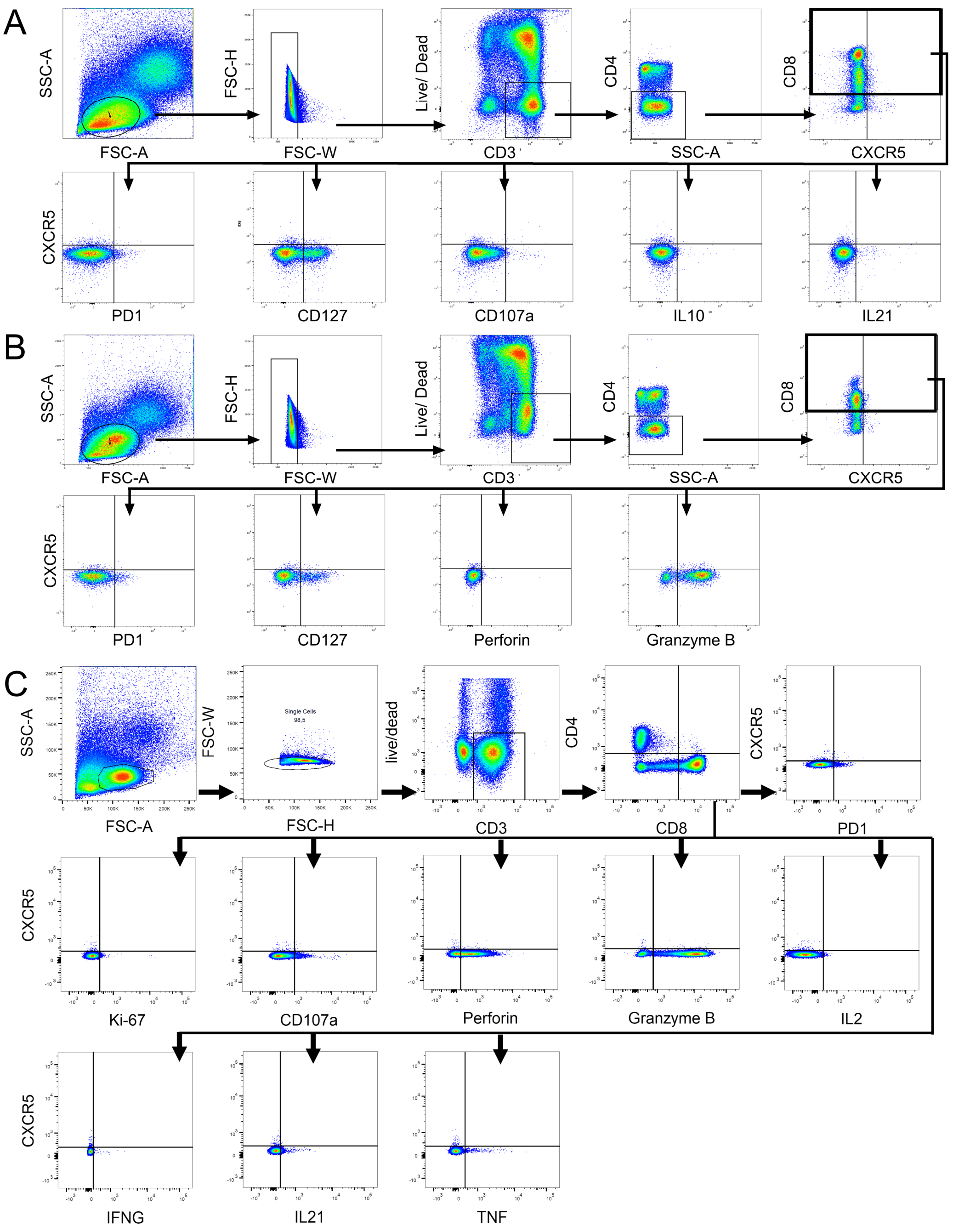

Supplemental Figure 9: Exemplary gating strategy for intracellular cytokine staining assays measured by flow cytometry.

PBMCs were stimulated O/N with a gag peptide pool **(A)**, kept unstimulated **(B)** or additionally treated with a PD1-blockade (sample without PD1 blockade is depicted) **(C)**.

Supplemental Table 2: Characteristics of participants for viral inhibition assay.

Participant	Sex	Age (years)[A]	CD4-count (cells/µl)[B]	Plasma viral load (copies/ml)[B]	Time diagnosis to sampling (months)[A]	Time between samples (months)
38	m	28	589	134,946	8	9
48	m	22	599	46,372	1	7
53	m	28	639	137,976	10	12

[A]as first blood sample was drawn; [B]mean value of the blood samples

Supplemental Table 3: Summary of main reagents and their supplier.

Reagent	Source	Concentration (µg/ml)	Used for…
Histopaque 1077	Sigma Aldrich	n.a.	PBMC processing
SL9 monomers	Provided by David A. Price	n.a.	Low input RNA-sequencing
RM9 monomers	ImmuneAware	n.a.	Low input RNA-sequencing
KL9 monomers	MBL International	n.a.	Low input RNA-sequencing
Zombie NIR™ Fixable Viability Kit	BioLegend	n.a.	Flow cytometry/cell sorting for RNA-sequencing
FIX & PERM Medium A&B	Thermo Fisher Scientific	n.a.	Flow cytometry
Rainbow beads	BD Bioscience	n.a.	Flow cytometry
HIV gag and nef	NIBSC	12.5	ELISpot
IFNG monoclonal antibody 1-D1K (#3420-3-250)	Mabtech	n.a.	ELISpot
SEB	Sigma Aldrich	12.5	ELISpot
FEC-peptide pool (CMV, EBV, Influenza peptides)	NIBSC	12.5	ELISpot
IFNG detection antibody 7-B6-1 (#3420-6-250)	Mabteck	n.a.	ELISpot
AP Conjugated Substrate Kit	Biorad	n.a.	ELISpot
Opal™ 4-Color Manual IHC Kit	Ayoka Biosciences	n.a.	Multiplex immunofluorescence microsopy
SuperScript II Reverse Transcriptase	Thermo Fisher Scientific	n.a.	Low input RNA-sequencing
KAPA HiFi HotStart ReadyMix	Roche	n.a.	Low input RNA-sequencing
Nextera XT DNA Library Preparation Kit	Illumina	n.a.	Low input RNA-sequencing
Nextera XT Index Kit v2	Illumina	n.a.	Low input RNA-sequencing
Agencourt AMPure XP beads	Beckman Coulter	n.a.	Low input RNA-sequencing
Qubit dsDNA HS Assay Kit	Thermo Fisher Scientific	n.a.	Low input RNA-sequencing
Agilent High Sensitivity DNA Kit	Aglient	n.a.	Low input RNA-sequencing
HIV SF2 overlapping peptide pools spanning gag proteins	NIBSC	2	Intracellular cytokine staining assays
SEB	Sigma Aldrich	1	Intracellular cytokine staining assays

Anti-CD28 antibody (#555726)	BD Bioscience	0.5	Intracellular cytokine staining assays
Anti-CD49d antibody (#555502)	BD Bioscience	0.5	Intracellular cytokine staining assays
Brefeldin A	Sigma Aldrich	10	Intracellular cytokine staining assays
GolgiPlug protein transport inhibitor	BD Bioscience	0.75	Intracellular cytokine staining assays
EasySep^{TM} Human CD4 T Cell Isolation Kit	StemCell Technologies	n.a.	Intact proviral DNA assay; viral inhibition assay
DNAzol^{TM}	Invitrogen	n.a.	Intact proviral DNA assay
Qiacuity Probe PCR Kit	Qiagen	n.a.	Intact proviral DNA assay
HIV p24 ELISA	Perkin-Elmer	n.a.	Viral inhibition assay
Gag-peptide pool (ARP-12437)	NIH HIV reagents program	0.5	PD1 blockade
Nivolumab (BMS-936558)	Bristol-Myers Squibb	10	PD1 blockade
IgG4-isotype control (clone QA16A15)	BioLegend	10	PD1 blockade

Supplemental Table 4: Composition of buffer and media prepared in the lab.

Buffer/medium	Composed of…	Used for…
Cryo medium	90% FCS, 10% DMSO	PBMC processing/storage
R10 medium	RPMI-1640 supplemented with 10% FCS, 1% L-glutamine, 1% Hepes, 1% penicillin/streptomycin	Cell culture
FACS buffer	PBS containing 1mM EDTA, 0.5% FCS or BSA	Cell sorting
Lysis buffer	0.2% Triton-X-100, 100µM Oligo dT primer, 10mM dNTP mix, 40U/µl recombinant Rnase Inhibitor (Takara Bio in molecular grad H2O)	Low input RNA-sequencing
R10-50	R10 with 50U/ml IL2	Viral inhibition assay
R10 containing ART	R10 medium supplemented with 18µM Azidothymidin, 10µM Nevirapine, 20µM Raltegravir	PD1 blockade

Supplemental Table 5: Devices.

**Device**	**Manufactor**	**Used for…**
Casy 1TT	SCHÄRFE SYSTEM	Cell counting
MoFlo Astrios Cell Sorter	Beckmann Coulter	Cell sorting
BD LSRFortessa^{TM}	BD Bioscience	Flow cytometry
AID ELISPOT reader ELR04	Autoimmun Diagnostika GmbH	ELISpot
Shandon Finesse 325 rotary microtome	Thermo Fisher Scientific	Mulitplex immunofluorescence microscopy
Axio Z2 microsope	Zeiss	Mulitplex immunofluorescence microscopy
HiSeq^{TM}	Illumina	Low input RNA-sequencing
Novaseq^{TM}	Illumina	Low input RNA-sequencing
Quantus^{TM} Fluorometer	Promega	Intact proviral DNA assay
Qiacuity One 5-plex digital PCR instrument	Qiagen	Intact proviral DNA assay

Supplemental Table 6: List of antibodies used for flow cytometry and multiplex immunofluorescence microscopy.

Antigen	Fluorochrome	Clone	Source	Identifier	Pheno	ICS	Sorting for RNAseq	Sorting for viral inhibition assay	PD1 Block	Confirmation of selected DEGs	Immunofluorescence microscopy
CCR7	BV 650	G043H7	BioLegend	353233 353234	x						
CD107a	BV 785	H4A3	BioLegend	328644		x			x		
CD127	PE/Cyanine 5	R34.34	Beckman Coulter	A64617	x	x			x		
CD161	BV 421	HP-3G10	BioLegend	339913	x						
CD27	PE	M-T271	BioLegend	356406			x				
CD3	BV 510	OKT3	BioLegend	317332	x	x	x	x	x		
CD39	BUV 737	TU66	BD Bioscience	564726	x						
CD4	PerCP/Cyanine 5.5	RPA-T4	BioLegend	300530	x	x	x	x	x		
CD45RA	BUV 395	HI100	BD Bioscience	740298	x						
CD45RA	BV 421	HI100	BioLegend	304130			x				
CD57	PE	NK-1	BD Bioscience	560844	x						
CD73	PE/Dazzle 594	AD2	BioLegend	344019	x						
CD8	AlexaFluor 700	HIT8a	BioLegend	300920	x	x	x	x	x		
CXCR5	AlexaFluor 488	RF8B2	BD Bioscience	558112	x	x	x	x	x		
Granzyme B	Pacific blue	GB11	BioLegend	515408		x			x		
HLA-DR	PE/Cyanine 7	3D12	BioLegend	342607	x						
IFNG	BUV 395	B27	BD Bioscience	563563		x			x		
IL10	BV 650	JES3-9D7	BD Bioscience	564051		x					
IL17A	APC	BL168	BioLegend	512334		x					

IL21	PE	3A3-N2	BioLegend	513004		x			x		
PD1	BV 605	EH12.2H7	BioLegend	329923	x	x			x		
Perforin	PE/Dazzle 594	dG9	BioLegend	308132		x			x		
TCR Vα7.2	BV 785	3C10	BioLegend	351721<br>351722	x						
TIM3	BV 711	F38-2E2	BioLegend	345024	x						
TNF	PE/Cyanine 7	MAb11	BioLegend	502930		x			x		
Ki-67	BV711	B56	BD Bioscience	563755					x		
IL2	APC	MQ1-17H12	BioLegend	500309					x		
CTLA	BUV 737	14D3	Invitrogen	367152941					x		
CD4	AF700	RPA-T4	BioLegend	300526						x	
CD3	BUV395	SK7	BD Bioscience	564001						x	
CD69	BUV737	FN50	BD Bioscience	612817						x	
CD8	BV480	RPA-T8	BD Bioscience	566121						x	
TLR2	FITC	TL2.1	BioLegend	309706						x	
HLADR	RB705	G46-6	BD Bioscience	756955						x	
TLR5	PE	S16021I	BioLegend	394504						x	
CXCR5	PE-Cy7	J252D4	BioLegend	356924						x	
CD172a	APC	15-414	BioLegend	372106						x	
CD38	BUV496	HIT2	BioLegend	612946						x	
TLR8	BV421	S16018A	BioLegend	395510						x	
CXCR5	/	EPR23463-30	Abcam	ab254415							x
CD8	/	C8/144B	Agilent Technologies	M710301-2							x
HIV-p24	/	Kal-1	Agilent Technologies	M085701-8							x

Supplemental Table 7: Primers used for intact proviral DNA assay.

Primer/probe	Sequence
Env-MA f	AGTGGTGSAGAGAGAAAAAAGAGC
Env-TV p V3	AA+G C+AC T+A+T +GGG C
Env-TV p comp.	A+GC +ACT +AT+A +AGC GC
Env-MA r	CTGGCCTGTACCGTCAGCG
Psi-MA f	GCAGGACTCGGCTTGCTG
Psi-MA p	CGACTG+GTGA+GTACGCC
Psi-MA r	CACCCATCTCTCTCCTTCTAGCC
RPP30 fwd	GATTTGGACCTGCGAGCG
RPP30 probe	CTGACCTGAAGGCTCT
RPP30 rev	GCGGCTGTCTCCACAAGT
RPP30d1 fwd	AGAGAGCAACTTCTTCAAGGG
RPP30d1 probe	CCCGGCTCTATGATGTTGTTGCAGT
RPP30d1 rev	TCATCTACAAAGTCAGAACATCAGA

"+" indicates usage of locked nucleic acids (LNAs)

Supplemental Table 8: Sex as biological variable.

Group	Frequency of male participants[A]
PLWOH	40% (4/10)
LTNP	60% (12/20)
TopHIVFUTURE	96% (26/27)
TopHIVPAST	100% (10/10)
Chronic	90% (9/10)
Tonsils	0% (0/4)

[A]in brackets: ratio of male to total participants
